# The adenine-modified edible chitosan films containing choline chloride and citric acid mixture

**DOI:** 10.1038/s41598-023-39870-4

**Published:** 2023-08-03

**Authors:** Magdalena Gierszewska, Ewelina Jakubowska, Agnieszka Richert

**Affiliations:** 1 Chair of Physical Chemistry and Physicochemistry of Polymers, Faculty of Chemistry, Nicolaus Copernicus University in Toruń, 7 Gagarina Street, 87-100 Toruń, Poland; 2 Faculty of Biological and Veterinary Sciences, Nicolaus Copernicus University in Toruń, 1 Lwowska Street, 87-100 Toruń, Poland

**Keywords:** Polysaccharides, Sustainability, Biopolymers

## Abstract

A series of biopolymeric chitosan-based (Ch) films were prepared with choline chloride and citric acid plasticizer (deep eutectic solvent, DES). An effect of adenine (A, vitamin B4) addition on the functional properties of these films was evaluated. Several physicochemical and mechanical properties were tested: Fourier-transformed infrared spectra proved DES's plasticizing and crosslinking effect, while scanning electron microscopy and atomic force microscopy techniques confirmed the possible phase separation after adenine addition. These changes affected the mechanical characteristics and the water vapor and oxygen permeability. The prepared materials are not water soluble because the CA acts as a crosslinker. The adenine addition on antioxidative and antimicrobial properties was also checked. It was found that Ch-DES materials with A exhibit improved antioxidative properties (55.8–66.1% of H_2_O_2_ scavenging activity) in contrast to the pristine chitosan-DES material (51.1% of H_2_O_2_ scavenging activity), while the material is still non-mutagenic (lack of growth of *Salmonella typhimurium*) and possesses antimicrobial features (no *E. coli* observed for all the tested films and inhibition zones noted for *S. aureus*). The mentioned properties, reduced oxygen transmission (1.6–2.1 g m^−2^ h^−1^), and mechanical characteristics within the range of typical food packaging plastics proved the potential of Ch-DES-A films in the packaging sector. Moreover, the antioxidative properties, usage of substrates being allowed as food additives, and the presence of adenine create the advantage of the Ch-DES-A materials as edible coatings, being also a source of Vitamin B4.

## Introduction

For several years there has been an increasing interest in exchanging non-biodegradable synthetic polymers applied in different industry sectors with biodegradable ones and those received from renewable resources. Biodegradable polymers degrade naturally and are composted by microorganisms, and the products of that process constitute valuable and safe substances returned to the environment^1^. Even if the biopolymers’ application is more expensive in many cases, their usage beneficially affects the natural environment and reduces the volume of polymeric wastes. What is crucial from the green-chemistry and ecological point of view, the replacement mentioned above should result in the reduction of the financial cost borne for the removal or recycling of conventional plastics wastes. Additionally, the biopolymer's degradation products will constitute the added value to stabilizing the environment by reducing carbon dioxide emissions and possibly using the produced CO_2_, water, biomass, humic matter, and other natural chemicals in the plant and animal life cycle.

Biodegradable polymeric materials are widely described in the literature. The most common in scientific articles are research on polymers such as polylactide (PLA), poly(ε-caprolactone) (PCL), or poly(3-hydroxybutyrate)—P(3HB), starch, cellulose and chitosan^2–7^. These materials are commonly suggested as substituents for the production of packaging films that form an integral part of the finished product and make the product attractive. Today, however, packaging should meet more and more different requirements. In addition to its essential functions, it must be environmentally friendly and readily biodegradable after use. Moreover, it should bring additional features, like being edible or possessing improved antioxidant or antimicrobial activity^3,8,9^. Such packages are classified as "active packages"^10^.

So far, the main limitation of using these new materials has been the high price and fragility, flexibility, or too little protection against undesirable microorganisms compared to classic synthetic materials. To eliminate these defects, biodegradable materials are modified, including the introduction of plasticizers and bioactive substances into the polymer matrix to protect the packed product against the harmful effects of microorganisms, i.e., to show a biocidal or at least biostatic nature^11–13^.

Among different biodegradable polymers, chitosan constitutes a highly prospective material as it exhibits desirable features (non-toxicity, biodegradability, biocompatibility, etc.) and can also be used as an edible antimicrobial package^14,15^. Films made of chitosan are rigid and fragile. Thus for several years, scientists have focused on the novel class of additives acting as chitosan plasticizers and, in some cases, as a crosslinker, namely deep eutectic solvents (DESs)^15–17^. The previously performed research^18–20^ proved the high potential of several DESs in improving chitosan films' elasticity. The undoubted advantage of DESs in this area is their "green solvents" classification, as most DESs are based on renewable, cheap, non/low-toxic, and biodegradable components, such as sugars, carboxylic acids, polyols, amines, and others. Additionally, the physicochemical properties of this type of mixture can be adjusted simply through the changes in the component type and their molar ratio^16^. It was also found that the addition of DESs affects numerous physicochemical features of polymeric films and enhances their potential in food packaging applications. As some of the Ch-DES materials have also shown self-healing features^21^, their usage as packaging contributes to overall food safety in storage and transport.

Among different DESs used as plasticizers for polymeric film production choline chloride (ChCl)-citric acid (CA) mixture can also be found. The number of research devoted to this system is, however, negligible. Smirnov et al.^21^ prepared self-healing Ch–ChCl–CA films with DES content between 62 and 82 wt.%, while the film with 67 wt.% of DES had the most homogeneous internal structure. Galvis-Sánches et al.^22^ developed Ch–ChCl–CA of constant chitosan-DES mixing ratio equal 70/30 (w/w), but films were fabricated by thermo-compression molding. Chitosan thermo-compressed films incorporating ChCl-CA exhibited excellent properties compared to the other applied DESs, and, thus, a high potential to be tested for food packaging applications. Song et al.^23^ obtained a novel antimicrobial packaging film using chitosan, ChCl–CA DES (a plasticizer), and tea tree essential oil (TTO). TTO reduced the hydrophilicity of the films, and DES significantly improved the flexibility. The prepared films showed the characteristics required of active packaging materials. ChCl–CA DES has also been used for the formation of other polymer-based systems: cellulose nanofibrils via choline chloride-citric acid DES pretreatment combined with high-pressure homogenization^24^, nanocellulose using acidic DESs based on choline chloride and carboxylic acids^25^.

The current trends in modification of chitosan-DES-based materials are directed toward making the material more attractive through the: (a) improvement of the antioxidant and antimicrobial properties, (b) reduction of undesirable high water vapor permeability. Several researchers analyzed the effect of different additives on the antimicrobial properties of the two-component chitosan-additive materials. Among various compounds added to Ch, numerous types of essential oils (EO: clove, carvacrol, oregano, and lemongrass, etc.), antibiotics, antimicrobial peptides, natural compounds, metals (gold, silver, or copper) can be found^15,26,27^. A similar attempt has also been made for various Ch-DES materials^28,29^.

The literature indicated only one research^23^ devoted to preparing the chitosan films plasticized with a choline chloride-citric acid mixture with improved flexibility and antimicrobial properties. The addition of TTO caused the reduction of water vapor permeability and potent antibacterial effects against *Sh. flexneri*, which implied that the film could prevent food contamination caused by foodborne bacteria. As a further development of food packaging based on chitosan films requires the invention of a new biomaterial-based composite, combining good mechanical, antioxidant, and antimicrobial properties, thus this study aimed modification of the physicochemical features of chitosan/choline chloride-citric acid films by using adenine (A). The hypothesis regarding adenine acting as a factor limiting the growth of pathogenic bacteria and antioxidative stress has been stated. Adenine, also known as a vitamin B4, is a purine nucleobase with an amine group at position C6; one of four chemical bases found in DNA, along with cytosine (C), guanine (G), and thymine (T). Adenine is also a component of adenosine, among which adenosine triphosphate (ATP) constitutes an energy molecule essential to vital human processes and functions. Vitamin B4 also possesses other essential functions: it promotes cell formation and, thus, tissue development, boosts the immune systems, and may, at some level, act as an antioxidant. Deficient of Vitamin B4 can cause several disorders (skin, blood, immune system, etc.), and incorporation of adenine into the edible package can alleviate chosen symptoms of different diseases (e.g., Alzheimer's Disease, Parkinson's, heart palpitation, gout, infection with bacteria and viruses, faster wound healing, anemia, arteriosclerosis, insomnia, allergies, cancer)^30,31^. To the best of the current knowledge, three-component systems based on chitosan-DES-adenine are obtained and tested for the first time.

## Materials and methods

### Materials and solutions

The chitosan powder (Ch) with a degree of deacetylation (DD) equal to 83.4 ± 2.4% applied for films' formation was acquired from BioLog Heppe GmbH (Landsberg (Germany)). Choline chloride (ChCl) (Acros Organics, Poland, 139.62 g·mol^-1^) and citric acid (CA) (Sigma Aldrich, Poland, 192.12 g mol^−1^) were used as received. An acetic acid solution of 2% (w/v) was prepared using concentrated acetic acid (Avantor Performance Materials Poland S.A, purity > 99%). Adenine (A) powder of 135.13 g·mol^-1^ molecular weight was purchased from Sigma-Aldrich and used as received. Deionized water was used throughout the entire experiment. The chemical structures of Ch, ChCl, CA, and A are given in Figure S1 (Supplementary Materials).

DES was prepared according to the following experimental procedure: choline chloride, previously dried at 70 ºC, acting as the hydrogen bond acceptor (HBA), was mixed with the appropriate amount of citric acid (hydrogen bond donor, HBD) at a 1:1 molar ratio, then the mixture was heated at 95 ºC under stirring until a clear liquid of DES (ChCl–CA) was reached. Afterward, the resulting DES was maintained at room temperature for 2 h before use.

Chitosan stock solution was prepared by dispersing chitosan in 2%(w/v) acetic acid at room temperature. The obtained solution was filtered and degassed.

Adenine stock solution was prepared by dissolving adenine in 2% (w/v) acetic acid to reach 0.01 g·ml^-1^.

### Film-forming solutions

The film-forming solutions were prepared according to a previously developed procedure^18,20^ by mixing DES, adenine, and chitosan solutions and vigorously stirring at room temperature for 24 h. The chitosan-DES weight ratio was constant for all the formulations (40/60 (w/w)), while the percentages of the adenine varied between 0 and 3 wt.%, relative to the mass of Ch-DES.

### Film characterization

Most applied characterization methods were performed according to the previously developed and used for other chitosan-based materials^18–20^.

#### Fourier-transformed infrared spectroscopy

FTIR spectra of all neat components and polymeric films were recorded using FT-IR Vertex 70 V (Bruker Optik) with the diamond crystal in ATR mode. The following recording parameters were used: resolution 4 cm^−1^, number of scans 32, wavelength range 400–4000 cm^−1^.

#### SEM and AFM imaging

Scanning electron microscopy (SEM) surface and cross-section imaging was performed using the LEO1430 VP microscope (Leo Electron Microscopy Ltd., Cambridge, UK). Dry samples were immersed in liquid nitrogen and cracked into small pieces before testing. A thin layer of gold was sputtered to improve surface layer conductivity.

Surface roughness and morphology of thin films were analyzed at room temperature in the air using a microscope with a scanning SPM probe of the NanoScope MultiMode type (Veeco Metrology, Inc., Santa Barbara, CA, USA), which operated in a tapping mode. Film samples of 1 × 1 cm^2^ were cut and subjected to analysis. Surface roughness was determined by measuring the root-mean-square (Rq) roughness and the arithmetic mean (Ra) roughness parameters within the Nanoscope v6.11 software (Bruker Optic GmbH, Ettlingen, Germany).

#### Mechanical properties

Elongation at break (*E*_*b*_), tensile strength (*TS*), and Young’s modulus (*YM*) were determined using an Instron 1193 testing machine equipped with adequate tensile test grips according to the PN-C-89034:1981 with the crosshead speed 1 cm·min^-1^. There were at least 10 repetitions per sample.

The thickness of the films (*h*) was measured in ten different film zones using a micrometer (Absolute Digimatic Indicator, Sylvac S229 swiss, Switzerland) with a precision of 0.001 mm.

The thickness values were used to determine the materials' density. For this purpose, several round-shaped samples were prepared using a circle cutter of known radius (*r*). The film density was determined by the sample weight and volume $\left( {\pi \cdot r^{2} \cdot h} \right)$.

#### Color measurements

The CIELab color values (*L, a, b*) were recorded (Dr. Lange MICRO-COLOR II LCM 6 colorimeter) and used for color evaluation. *L* (lightness: 0 = black, 100 = white); *a* (− *a* = greenness, + *a* = redness); and *b* (− *b* = blueness, + *b* = yellowness) values were the basis for the calculation of total color difference (*ΔE*), chroma (C*) and hue angle (*Hue*) according to the Eqs. (1)–(5)^32^:

1 $$\Delta E= \sqrt{{\Delta L}^{2}+{\Delta a}^{2}+{\Delta b}^{2}}$$

where *ΔL* = L − L*; Δa = a – a*; Δb = b − b* (L*, a*, and b* represent the standard values of the white calibration plate used as the background during film measurements).

2 $${C}^{*}= \sqrt{{a}^{2}+{b}^{2}}$$

3 $${Hue}= {tan}^{-1}\left(b/a\right)\mathrm{\,for\,} {a}>0\mathrm{\,and\,} {b}>0,$$

4 $$Hue= 180^\circ + {tan}^{-1}\left(b/a\right)\mathrm{\,for\,} {a}<0,$$

or

5 $$Hue=360^\circ + {tan}^{-1}\left(b/a\right)\mathrm{\,for\,} {a}>0\mathrm{\,and\,} {b}<0,$$

#### Opacity

Opacity was determined according to the Siripatrawan and Harte^33^ method. The films were cut into rectangular pieces and placed in a spectrophotometer test cell. An empty test cell was used as the reference. The film absorbance at 600 nm was measured using a UV–Vis spectrophotometer (Ruili Analytical Instrument Company, Beijing, China). Finally, the opacity of the films was calculated with the following Equation:

6 $$Opacity [{mm}^{-1}]= \frac{{A}_{600}}{h}$$

where: *A*_*600*_ is the absorbance at 600 nm, and *h* is the film thickness [mm]. According to this Equation, the high values of absorbance result in more opaque and less transparent materials.

#### Water vapor transmission rate (WVTR) and water vapor permeation (WVP)

The WVTR of films was investigated using the method described by Souza et al.^34^ with slight modifications. The tested films were sealed on the top of 29 mm diameter plastic containers containing a known mass of dried calcium chloride (0% relative humidity, RH) and placed at 30 °C in a desiccator containing a saturated NaCl solution (RH = 75%). The desiccant was prepared by drying at 110 °C under reduced pressure for 24 h before use. Five independent samples were prepared for each film. The amount of water vapor permeated was evaluated based on the changes in the weight of the container with the film, and CaCl_2_ was determined each 1 h. It was found that 24 h was enough to reach an equilibrium state. The values of WVTR were calculated according to the following Equation:

7 $$\mathrm{WVTR} [\mathrm{g}\cdot {\mathrm{m}}^{-2}\cdot {\mathrm{h}}^{-1}]=\frac{w}{A\cdot t}$$

where *w*—is the weight gained [g], *t*—time [h], and *A*—the area of the film exposed to water vapor permeation [m^2^].

As the films exhibited different thicknesses, also the WVP was calculated

8 $$\mathrm{WVP} [\mathrm{g}\cdot \mathrm{m}\cdot {\mathrm{m}}^{-2}\cdot {\mathrm{h}}^{-1}\cdot {\mathrm{Pa}}^{-1}]=\frac{\mathrm{WVTR}\cdot h}{\Delta Pv}$$

$\Delta Pv$ represents the partial pressure difference of the water vapor at test temperature between the two sides of the film (*P*_*container*_ = 0 Pa, *P*_*saturated NaCl*_ = 3218 Pa, $\Delta Pv$ = 3218 Pa at 30 °C).

#### Oxygen transmission rate (OTR)

The oxygen transmission rate (OTR) of polymer films was carried out by measuring the changes in oxygen content in the distilled water as a recipient using Winkler's method^35^. Deionized water was boiled for 15 min to remove dissolved oxygen, and then 50 ml was transferred to a plastic container (40 mm in diameter), finally, covered with polymer films. The open container, allowing oxygen to enter the flask and dissolve in the water freely, was used as a control. The flasks were placed in an open environment at room temperature for 24 h. The results were expressed as the amount of dissolved oxygen (*DO*). All studies were carried out in triplicate. The oxygen transmission rate (OTR) was then calculated:

9 $$\mathrm{OTR} [\mathrm{g}\cdot {\mathrm{m}}^{-2}\cdot {\mathrm{h}}^{-1}]=\frac{DO \cdot V}{A\cdot t}$$

where *DO*—is the dissolved oxygen [g dm^−3^], *V*—the volume of the water used [dm^3^], *t*—time [h], and *A*—the area of the film exposed for oxygen permeation [m^2^].

#### Antioxidant activity

##### DPPH radical scavenging assay

The antioxidant activity of the film samples was evaluated using DPPH (2,2-diphenyl-1-picrylhydrazyl) free radical scavenging assay according to Siripatrawan and Harte^33^ with slight modification. Briefly, 500 μl of methanolic extracts of chitosan films (2 g of solid/30 mL of MeOH) was introduced into the test tubes, followed by 2 mL of methanolic solution of DPPH (C = 304 μmol·dm^-3^). The test tubes were kept in the dark at room temperature, and the absorbance was measured at 517 nm. When the DPPH solution was mixed with the sample mixture, a stable non-radical form of DPPH was formed with a simultaneous change of the violet color to pale yellow. The percentage scavenging activity was calculated using the following Equation:

10 $$DPPH\, scavenging\, activity [\%]= \frac{{Abs}_{DPPH}- {Abs}_{extract}}{{Abs}_{DPPH}} \cdot 100$$

where: ${Abs}_{DPPH}$ is the absorbance of the methanolic solution of DPPH, and ${Abs}_{extract}$ is the absorbance of the sample extracts. Each sample was assayed at least five times.

##### Hydrogen peroxide radical scavenging assay

The hydrogen peroxide (H_2_O_2_) radical scavenging activity of chitosan films was determined using the method reported by Hafsa et al.^36^ Film extract was prepared by adding 500 mg film pieces into 15 ml methanol and ultrasonication of the mixture for 3 h, followed by 30 min centrifugation to collect the supernatant. 3 mL of fresh 40 mM H_2_O_2_ solution (in phosphate buffer pH 7.4) was mixed with 500 μL of different methanolic extracts, incubated at 37 °C for 10 min, and absorbance was measured at 230 nm. A phosphate buffer without H_2_O_2_ was taken as a blank. For each concentration, a separate blank sample was used for background subtraction. Antioxidant activity was determined using the Equation:

11 $${H}_{2}{O}_{2}\, scavenging\, activity [\%]=\left[1-\frac{{A}_{sample}}{{A}_{control}}\right]*100$$

where: *A*_*sample*_ is the absorbance of a mixture containing film extract and H_2_O_2_ solution, *A*_*control*_ is the absorbance of H_2_O_2_ solution without film extract.

#### Biological activity

##### Antibacterial properties

Antibacterial activity was determined based on the standard: ISO 20645, 2006 “Flat textile products. Determination of antibacterial activity. Diffusion method on an agar plate”^37^ Two bacterial reference strains were used in the study: *Escherichia coli* (ATCC 8739) and *Staphylococcus aureus* (ATCC 6538P).

The agar medium was inoculated with microorganisms, and a circular sample with a 25 ± 5 mm diameter was applied. Incubation was carried out for 20 h at 37 ± 1 °C temperature, and after that time, the zones of growth inhibition were measured and calculated using the following formula:

12 $$\mathrm{H}=\frac{D-d}{2}$$

where: H—braking zone width [mm], D—total diameter of the working sample and width of the braking zone [mm], d—diameter of the working sample [mm].

The assessment criteria described in standard^37^ were used to assess the effectiveness of the antibacterial effect of the films (Table S1 in Supplementary Materials).

##### Ames test

A mutagenicity test was performed to determine the direct effect of the test substance or its metabolites on the cell’s genotype. The potential mutagenicity of the test samples was analyzed using the in vitro method according to the Ames test. M9 minimal medium (${\text{Na}}_{{\text{2}}} {\text{HPO}}_{{\text{4}}}{\text{}}\times {\text{12 H}}_{{\text{2}}} {\text{O, KH}}_{{\text{2}}} {\text{PO}}_{{\text{4}}} {\text{, NaCl, NH}}_{{\text{4}}} {\text{Cl, agar, MgSO}}_{{\text{4}}} {\text{, CaCl}}_{{\text{2}}}$, glucose, ampicillin, biotin, histidine) was inoculated with Salmonella typhimurium strain. Then, the samples were placed on the prepared pans. The controls constituted Petri dishes containing only the medium with the Salmonella typhimurium inoculated strain. All plates were incubated upside down and wrapped in aluminum foil for two days at 37 °C. The lack of growth of a significant amount of bacterial cells around the sample indicates that the film is not mutagenic.

### Statistical analysis

All values are presented as mean ± standard deviation. To evaluate the significance of adenine addition on the changes of all the evaluated parameters, the analyses of variance (one-way ANOVA) were performed using SPSS/PS Imago Pro (version 8.0, Predictive Solutions, Poland). Differences were considered significant if the *p* < 0.05.

## Results and discussion

### FTIR spectroscopy

FTIR spectra of adenine (A), citric acid (CA), choline chloride (ChCl), and choline chloride-citric acid (1:1 mol/mol) deep eutectic mixture are provided in Supplementary Materials (Figures S2 and S3). In the adenine spectrum (Figure S2) following characteristic bands were noticed^38^: 1671 cm^−1^ (δ NH_2_), 1599 cm^−1^ (ν(C=N), ν(C=C)), 1416 cm^−1^ (δ(N=CH)), 1305 cm^−1^ (ν(C–N), ν(C=N)), 1249 cm^−1^ (ν(C–NH_2_)), 1159 cm^−1^ (δ(CH) in-plane), 1023 cm^−1^ (δ(C–N–C)), 937 cm^−1^ (δ(N–C=N)), 631 cm^−1^ (δ(N–C–C)). The 3600–2800 cm^−1^ bands represent symmetrical (3284 cm^−1^) and asymmetrical (3106 cm^−1^) stretching vibrations in the –NH_2_ group forming H-bonds. The FTIR spectra of CA, ChCl, and their equimolar deep eutectic mixture (Figure S3) have been discussed in detail by us earlier^20^. It was stated that the formation of hydrogen bonds between both DES components results in the shifting of stretching vibration bands at ca. 3300 cm^−1^ and vibrational bands of C=O and C–O (in the carboxylic group) characteristic of CA structure.

**Figure 1:**
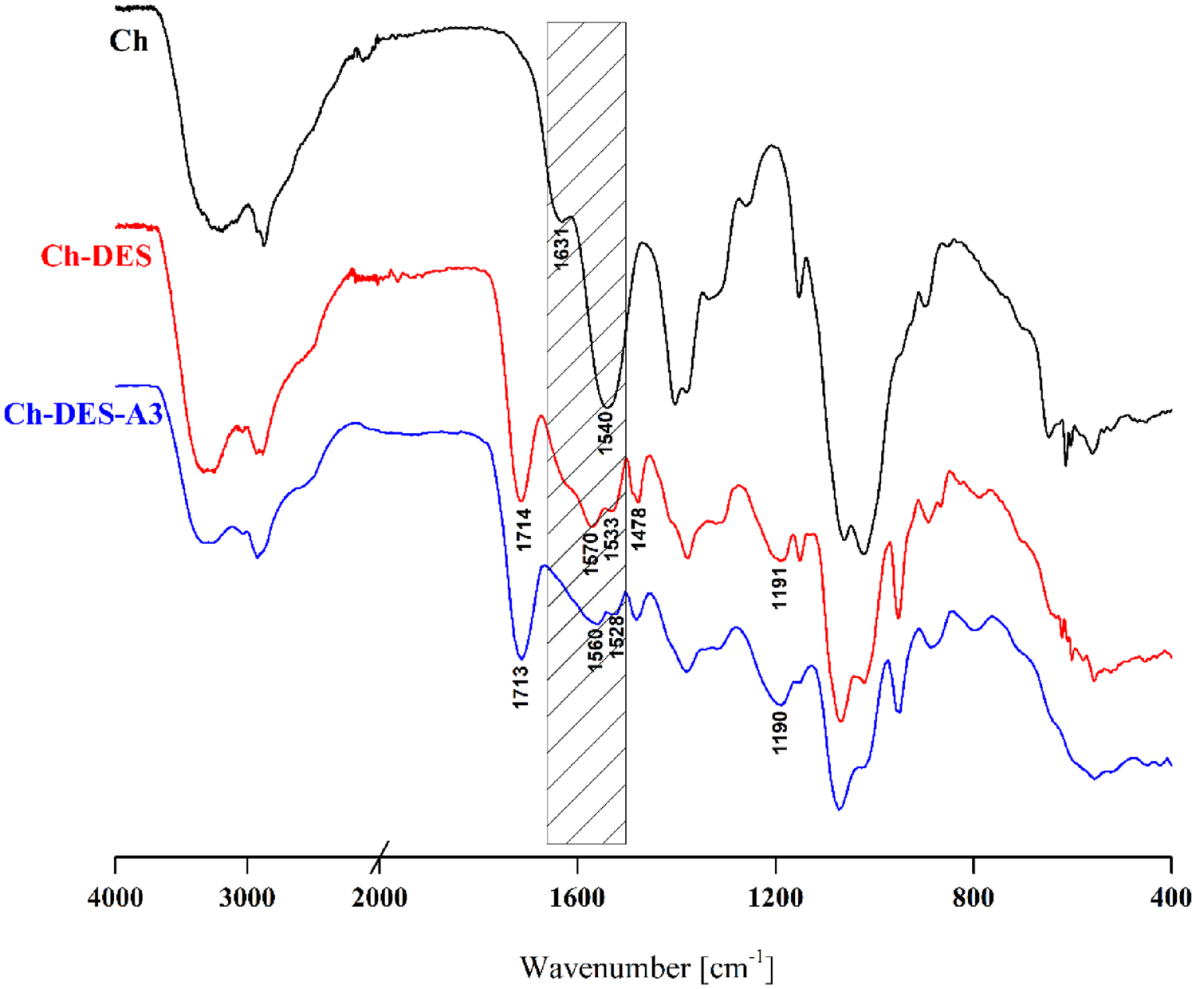
FT-IR spectra of Ch, Ch-DES, Ch-DES-A3 films.

The analysis of FTIR spectra recorded for Ch and Ch-DES films indicated several differences (Fig. 1). In the Ch-DES spectrum, new intense absorption bands, characteristic of the DES structure, were noticed^39^: (a) 1714 cm^−1^ (C=O vibration in carboxyl group), (b) 1478 cm^−1^ (CH_2_ bending), (c) 1191 cm^-1^ (C–O stretching). It can not be excluded that the band at 1714 cm^−1^ represents not only the vibration characteristic for CA but also the ester bonds formed in the reaction of –COOH groups in CA and –OH functionals of chitosan. As described by Wu et al.^40^, adding citric acid (CA) into the starch/chitosan (PS/CS) mixture resulted in a new peak at ca. 1724 cm^−1^ in the PS/CS/CA film spectrum. The authors stated that it could be attributed to esterification between CA and both polymers. Similar findings have been made by Seligra et al.^41^ for starch-based materials and Priyadarshi et al.^42^ for chitosan films plasticized with glycerol and crosslinked with citric acid.

Substantial changes also occurred in the spectral 1500–1650 cm^−1^ region, where the band at 1540 cm^−1^ corresponding to N–H bending in the amide group (amide II vibration) and the band at 1631 cm^−1^ representing C=O stretching in the amide group (amide I vibration) of chitosan were found^43^. After the DES addition, the broad vibrational band with two distinct submaxima (1570 and 1533 cm^−1^) and a small shoulder (1626 cm^-1^) in the spectrum of Ch-DES can be noticed. It is well known that amino groups of chitosan are protonated under acidic conditions and thus can ionically interact with anionic species. The existence of –NH_3_^+^ functionals in Ch-based materials can be confirmed by a new band in the FTIR spectrum located in higher frequencies than those of -NH_2_. Based on the obtained results, it can be assumed that within the Ch–ChCl–CA matrix, the new ionic crosslinking interactions between protonated amino groups of chitosan and -COO^-^ groups of citric acid occur. The changes in the vibrations corresponding to the amide II as a confirmation of ionic crosslinking were previously described by others^44,45^. The simple 24 h solubility test was also performed to prove the crosslinking process's occurrence. The neat Ch-film cast from the acetic acid solution disintegrated in water, while the Ch-DES and Ch–DES-A films retained their solid state. The effect of the ChCl-CA mixture on chitosan film solubility was already shown by us earlier^20^.

The differences between Ch and Ch-DES were also noticed in the 3000–3500 cm^−1^ region, which corresponds mainly to the vibrations of –NH_2_ and –OH. The bands observed in the Ch spectrum are in the spectrum of Ch-DES shifted into the higher frequencies. That indicated the changes in the hydrogen bonding after adding the ChCl–CA mixture to chitosan. It can be assumed that some H-bonds in chitosan are destroyed, but new ones between Ch, ChCl, and CA are formed.

In turn, there are no distinct differences between the Ch-DES and Ch–DES–A3 spectra. It is probably due to the low amount of adenine added concerning the amounts of other film components. Only slight shifting of some bands' positions can be noted. Thus, it can be assumed that in the applied preparation condition, the only interaction between adenine and other film constituents is the hydrogen bonding between –NH_2_ groups of A and –NH_2_/–OH groups of chitosan. It should also be mentioned that N atoms in the adenine ring structure, based on the calculated Mulliken partial charges^46^, can also act as H-bonds acceptors.

### Surface structure

**Figure 2:**
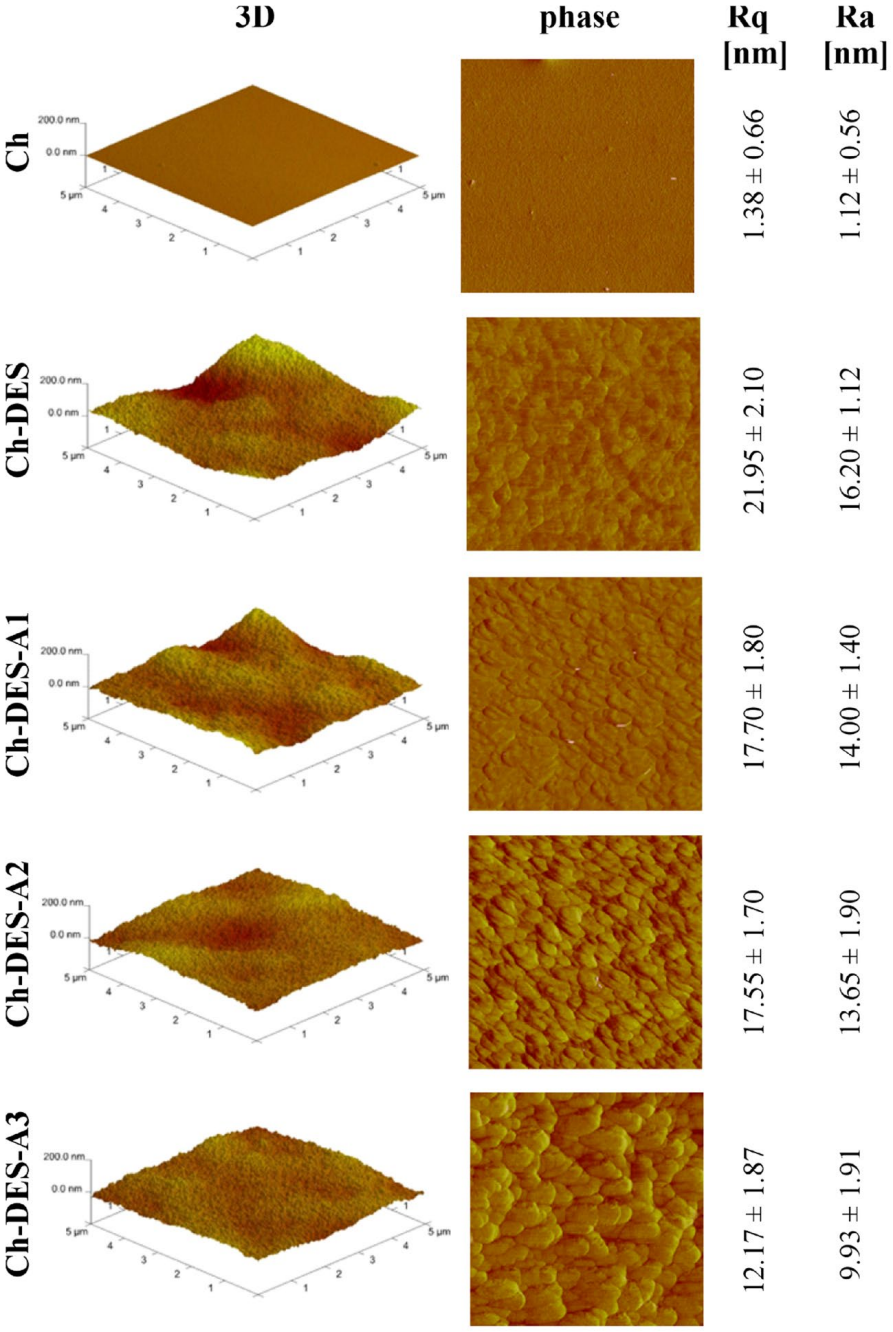
AFM 2D and phase images of chitosan films' surface and surface roughness parameters (Ra and Rq).

**Figure 3:**
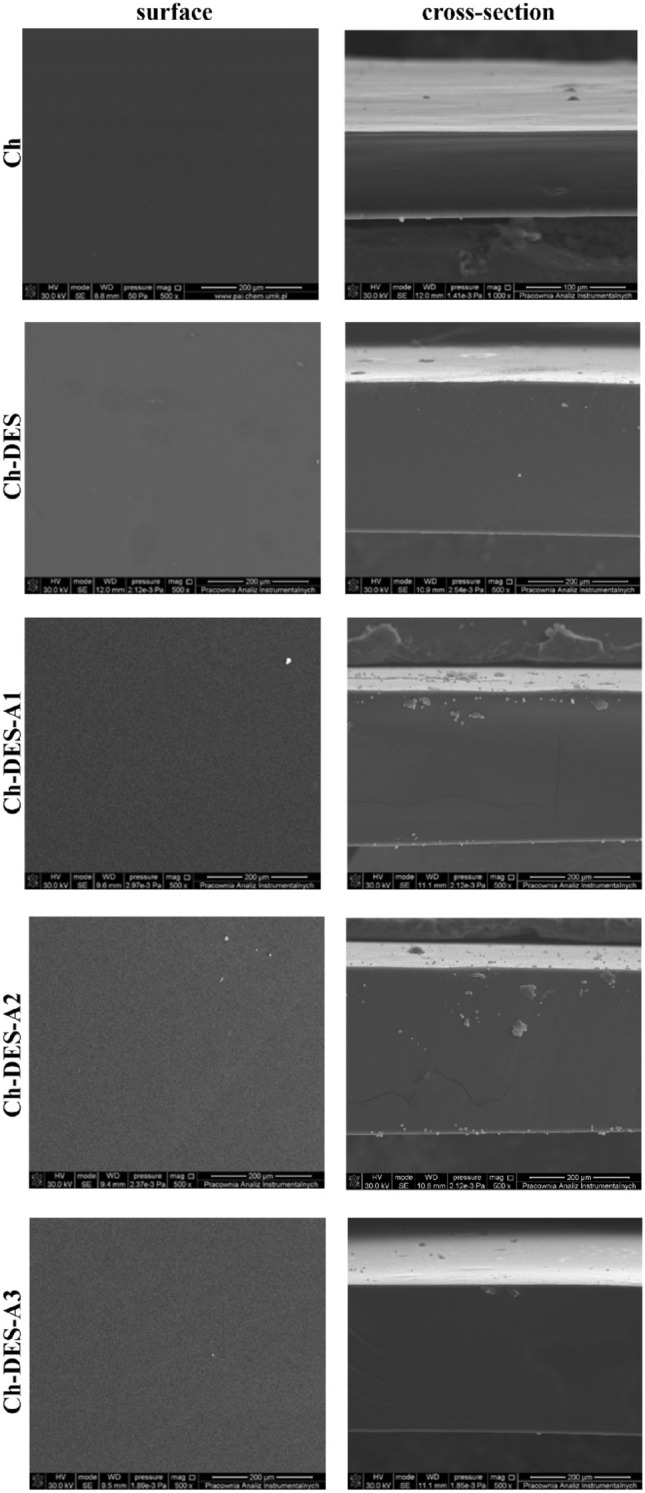
SEM images (500 × magnitude) of the surfaces and cross-section of the films.

AFM images show essential information about surface characteristics, including 3D topography images and quantitative data analysis (surface roughness, grain size, step height, etc.). At the same time, SEM imaging gives general specimen property information (compositional microstructure, topography, shape, etc.). Figure 2 represents three-dimensional and phase AMF images of neat and ChCl-CA modified chitosan-films with different adenine content. Simultaneously, the surface roughness parameters (Ra and Rq) are given. The SEM surface and cross-section images of these same materials are shown in Fig. 3.

The AFM and SEM images of the surface and fracture proved that all tested films are dense and non-porous. The most uniform and smooth surface was noticed for neat chitosan film. The effect of different DESs addition on the chitosan film surface morphology has been observed earlier by us^18–20^ and others^21,47,48^, especially for those materials where DES content exceeded 60 wt.%. The current findings, an increase in the Rq parameter from 1.38 ± 0.66 nm for neat chitosan film to 21.95 ± 2.10 nm for Ch-DES film, stay in agreement with the literature and can be explained the phase separation. As shown, for lower DES contents, DES penetrates the polymeric matrix and forms a continuous phase with the polymer. When DES content increases, the number of polymer functional groups is insufficient to interact with DES components. Thus, in the case of the Ch-DES film in the current study, the formation of micro intrusions can be suggested. Adding a low amount of adenine does not counteract the Ch-DES separation (Fig. 3, phase images). The AFM phase images revealed that with the increasing adenine content, the surface topography represents the larger nodules. As the size of the nodules increases, the apparent chitosan film roughness decreases, from 17.70 ± 1.80 nm for Ch-DES-A1 to 12.17 ± 1.87 nm for Ch-DES-A3. The higher size of nodules can be related to the chemical structure of adenine (Figure S1, Supplementary Materials). As the upper and lower surfaces of the adenine rings are hydrophobic, thus the addition of the nucleic acid acts synergistically with the DES addition on the chitosan-films surface structure, causing the progressive increase in phase separation phenomenon. Analogous changes in the surface morphology can be observed in the high-resolution SEM images (Figure S4a-e, Supplementary Materials).

### Mechanical properties

**Figure 4:**
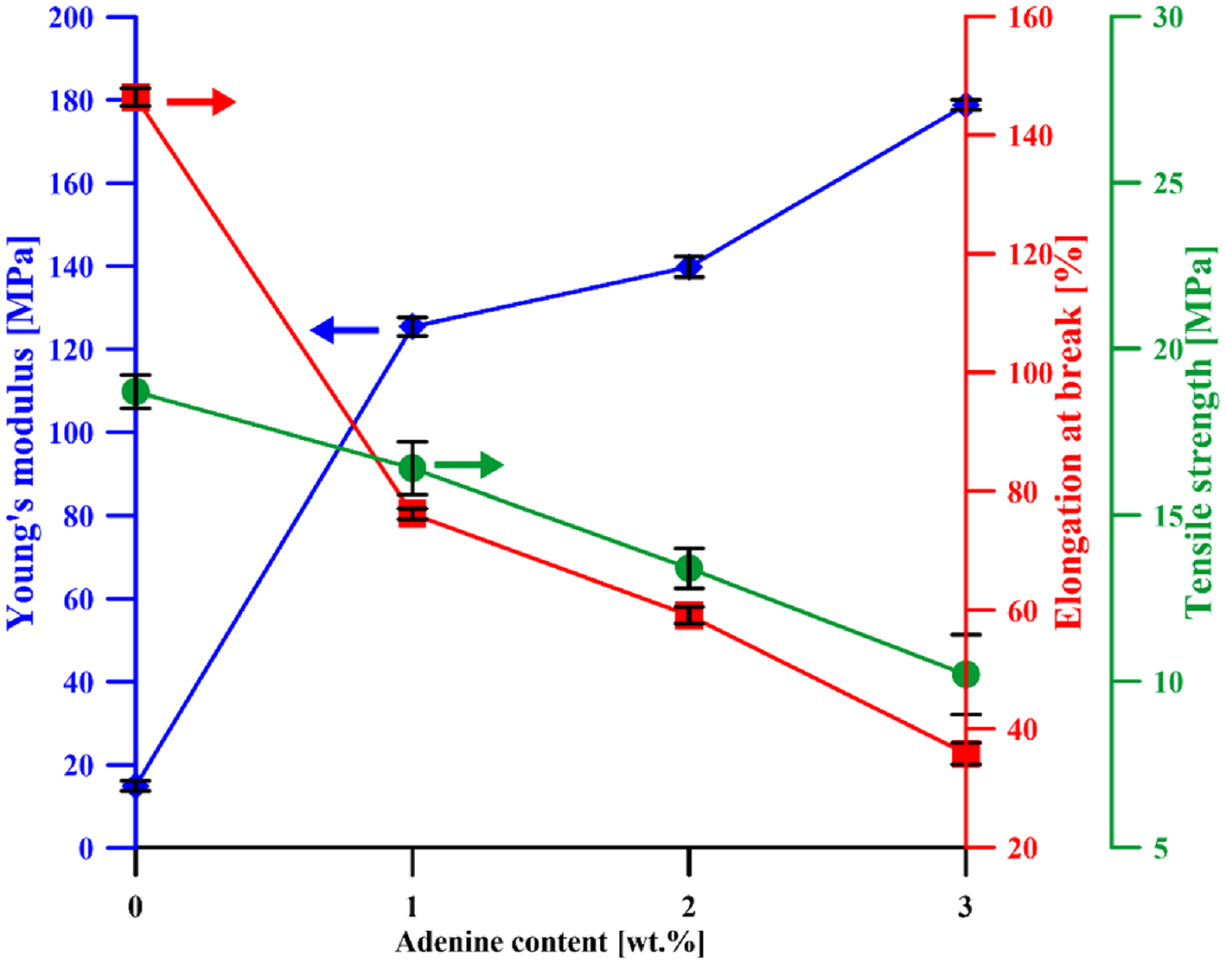
Evolution of Young's modulus (*YM*), tensile strength (*TS*), and elongation at break (*E*_*b*_) of Ch-DES films with different amounts of adenine.

The mechanical properties (Young's modulus, tensile strength, and elongation at break) of neat Ch-DES film and Ch-DES films with various amounts of adenine were compared (Fig. 4). The results showed that adenine significantly affected the mechanical resistance and extensibility of the chitosan-based films.

Elongation at the break (*E*_*b*_) is an indication of the films' flexibility and stretchability (extensibility), which is determined at the point when the film breaks under tensile testing and is expressed as the percentage of change of the original length of the specimen between the grips of a film to stretch (extend)^32^. The *E*_*b*_ values of the chitosan films decreased from 146.4 ± 1.5% for neat Ch-DES film to a minimum of 35.8 ± 1.8% when the adenine content reached 3 wt.% (Fig. 4). It can be assumed that this behavior is associated with the interactions of adenine with the Ch and DES components discussed in the FTIR section. As stated by Huang et al.^49^, hydrogen bonds, ionic, metal–ligand, host–guest, and hydrophobic interactions affect the mechanical properties of polymeric materials; however, this impact can be opposed. It was also found that this impact depends on the binding energy in the case of H-bonds. Namely, H-bonds of moderate binding energies can presumably improve the stretchability by delaying fracture or increasing the rigidity while high energy H-bonding (–COOH groups) is formed. In the case of multicomponent films being an object of this study, the overall changes (disrupting of existing H-bonds in Ch-DES, formation of new H-bonds with adenine) cause the formation of less flexible materials. Similar findings have been made by Ojagh et al.^50^ for chitosan films plasticized with glycerol containing a cinnamon essential oil (CEO). They noticed a substantial reduction in *E*_*b*_ after adding a low amount of hydrophobic CEO. Even if a significant reduction of elasticity was detected, the *E*_*b*_ values for Ch-DES-A films are still higher than this of pristine chitosan film^51^.

The tensile strength (*TS*) of the chitosan-based composite films behaved similarly to the *E*_*b*_ value, i.e., decreased with the increasing content of adenine. The tensile strength of a material is the maximum tensile stress sustained by the sample during the tension test, expressed as the maximum amount of tensile stress that it can take before failure (breaking or permanent deformation). Pure Ch-DES film exhibited a *TS* of 18.7 ± 0.5 MPa, while the TS of Ch-DES composite films with 1, 2, and 3 wt.% adenine decreased to 16.4 ± 0.8, 13.4 ± 0.6, and 10.2 ± 1.2 MPa, respectively, and are also lower than *TS* of neat chitosan film^51^. The observed trend indicates that the adenine causes the reduction of force needed to break the plasticized film. Thus it can be assumed that Ch-DES-A has a less ordered structure than Ch-DES film^52^.

Contrary to the *E*_*b*_ and *TS* changes, *YM* increases with the amount of the additive. The higher the adenine content, the more stiff the films are.

The literature data indicate that polymers most commonly used in the food packaging sector exhibit different mechanical properties, depending on the exact application area, especially the type of packaged food. Some packages are expected to be flexible, i.e., films, thin layer laminates, or rigid, i.e., thick plastics. As presented by Robertson^53^, there is considerable differentiation in mechanical characteristics between packages made with different polymers like PLA, PHA, PHB, PET, PS, PP, or LDPE. The TS values of the Ch-DES and Ch-DES-A films are within the range characteristic of polymers most commonly used in food packaging^53,54^, i.e., ca. 10 MPa for LDPE, through ca. 20 for PHA and 40 for PHB, up to ca. 70 for PET. Moreover, the Ch-DES and Ch-DES-A films possess acceptable elongation at break values, similar to various polymers used in food packaging, like PHA (*E*_*b*_ ca. 25%), PLA (*E*_*b*_ ca. 30–240%) or LDPE (*E*_*b*_ > 100%).

### Opacity and color

**Figure 5:**
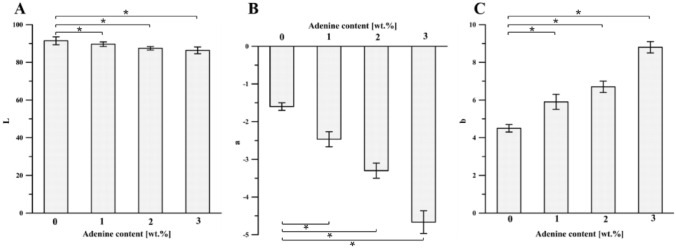
Effect of adenine content on (**A**) *L*, (**B**) *a*, and (**C**) *b* values of the Ch-DES films (values with asterisks * represent significant differences (*p* < 0.05) relative to the Ch-DES).

**Figure 6:**
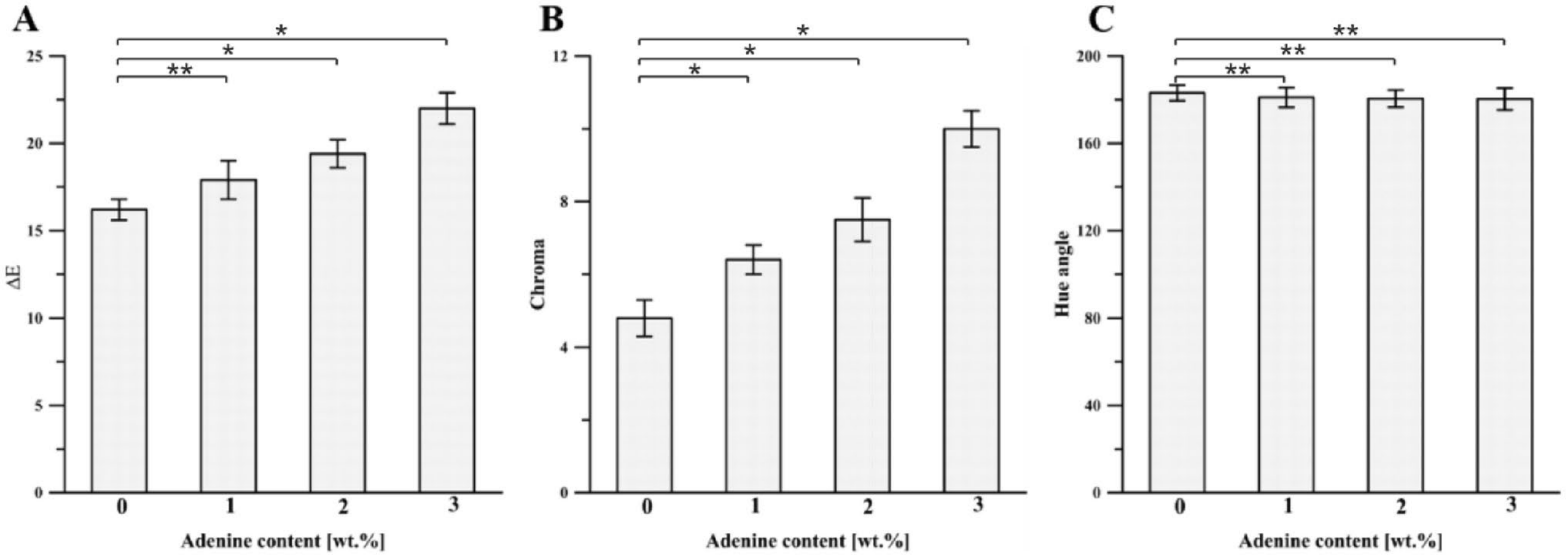
Effect of adenine content on (**A**) *ΔE* and (**B**) chroma values of the Ch-DES films (values with asterisks * represent significant differences (*p *< 0.05) while ** represents no significant differences (*p* > 0.05) relative to the Ch-DES).

Color properties are essential for film appearance, influencing consumer acceptance of the packaged products. The results of the measurements performed on the chitosan film's color were expressed following the CIELab system, and the rectangular coordinates (*L, a*, and *b*), the total color difference (*ΔE*), hue angle (*Hue*), and chroma were calculated (Figs. 5 and 6). The introduction of adenine into chitosan-DES films significantly affected *L* (lightness/darkness), *a* (redness/greenness), and *b* (yellowness/blueness) values of the film surface. It was found that with the higher adenine content, the darker films were obtained as indicated by *L* values (Fig. 5A), changing from 91.43 ± 2.11 (for Ch-DES film) to 86.37 ± 1.22 (for Ch-DES-A3). Moreover, also other color parameters change continuously: *a* decreased from -1.61 ± 0.11 to -4.67 ± 0.31 (an indicator of the tendency towards redness), while *b* values increased from 4.52 ± 0.20 to 8.84 ± 0.32 (an indicator of the tendency towards yellowness), as the adenine concentrations increased from 0 to 3 wt.% (Fig. 5B, C). According to the ANOVA analysis (Figs. 5 and 6, Table S2 in Supplementary Materials), all observed changes are statistically significant. Moreover, the high values of *ΔE* (Fig. 6A), calculated concerning the neat chitosan film, indicate that human eyes can notice the color difference. As *Hue* and C* are computed using *a* and *b*, they are indexed somewhat analogous to color saturation or intensity. The C* values increased when the adenine content also increased (Fig. 6B). It can be attributed to the characteristic of pure adenine provided by supplier^55^, who indicated the color of adenine as a light yellow. It was found that the values of *Hue* (Fig. 6C, Table S2 in Supplementary Materials) do not differ significantly within the applied adenine content.

Based on the opacity values calculated with Eq. 6 (Table 1), it can be stated that chitosan films without adenine were more transparent (lower opacity value) than those incorporated with adenine. The opacity of the film samples significantly increased with increasing adenine concentration. Ch-DES-A3 film has the highest opacity (0.583 ± 0.015) value, followed by Ch-DES-A2 (0.223 ± 0.019), Ch-DES-A1 (0.160 ± 0.013) and Ch-DES (0.039 ± 0.008) films. The increase in the opacity stays in agreement with the AFM analysis and can be explained by the light scattering occurring when different phases are present in the polymeric material.

**Table 1:** Opacity, thickness, density, water vapor transmission rate, and oxygen permeability of the Ch-DES and Ch-DES-A films.

Adenine content [wt.%]	Opacity $[{\mathrm{mm}}^{-1}$]	Thickness [cm]	Density [g∙${\mathrm{cm}}^{-3}]$	WVTR $[\mathrm{g }{\mathrm{m}}^{-2} {\mathrm{h}}^{-1}]$	WVP [10^–6^ g m m^-2^ Pa^-1^ h^-1^]	OTR $[\mathrm{g}\cdot {\mathrm{m}}^{-2}\cdot {\mathrm{h}}^{-1}]$
0	0.039 ± 0.008	0.0269 ± 0.0010	1.204 ± 0.019	15.1 ± 0.3	1.3 ± 0.3	3.4 ± 0.2
1	0.160 ± 0.013*	0.0349 ± 0.0007*	1.513 ± 0.023*	37.1 ± 2.9*	4.1 ± 0.3*	2.1 ± 0.3*
2	0.223 ± 0.019*	0.0361 ± 0.0017*	1.547 ± 0.022*	33.0 ± 1.8*	3.7 ± 0.2*	1.8 ± 0.4*
3	0.583 ± 0.015*	0.0374 ± 0.0021*	1.624 ± 0.031*	30.9 ± 1.1*	3.6 ± 0.1*	1.6 ± 0.7*

Values with asterisks * represent significant differences (*p* < 0.05) relative to the Ch-DES.

### Water vapor (WVTR) and oxygen transmission rate (OTR)

Water vapor and oxygen transmission properties are essential to food packaging materials. The permeation of oxygen and water from the environment to food has a crucial effect on food quality and shelf life. Oxygen causes food deterioration relying on lipid and vitamin oxidation, leading to sensory and nutrient changes. Thus, one of the main functions of a film for food packaging is to impede moisture and O_2_ transfer between food and the surrounding atmosphere, so the WVTR and OTR of the film should be as low as possible. As it is well known, the permeability of a film depends on its chemical structure and morphology, the nature of the permeant, and the temperature of the environment^56^.

The WVP of the chitosan films incorporated with different concentrations of adenine was examined at 30 °C (RH = 75%). The effect of adding ChCl-CA mixture into a neat chitosan matrix was discussed by us earlier^20^ and showed that DES addition increases WVTR by 75% in relation to neat Ch. It was ascribed to the hydrophilic characteristic of DES and its plasticizing role, resulting in improved segmental polymer chain mobility. The results of water transport properties through adenine-incorporated Ch-DES (Table 1, Table S2 in Supplementary Materials) showed that adenine also significantly affects the WVTR and WVP of the films. It was found that the addition of adenine substantially increases the WVTR and WVP parameters, but when the adenine content in Ch-DES film increases from 1 to 3 wt.%, the WVTR, and WVP slightly decrease from 37.1 ± 2.9 to 30.9 ± 1.1 g m^−2^ h^−1^, and from 4.1 10^–6^ to 3.6 10^–6^ g m m^-2^ Pa^−1^ h^−1^, respectively. Thus it can be stated that undesirable changes in WVTR and WVP values after DES addition are enhanced. The substantial increase in both water vapor transport parameters after adding adenine stands in opposition to the increase in density. However, it should be emphasized that in the case of films being an object of this study, adding adenine noticeably changed the internal structure of the chitosan-DES-based films discussed in the SEM and AFM section. Thus, it can be assumed that the enhancement of water vapor transport relates to the phase separation phenomenon. The slight decrease in WVTR and WVP with the increase in adenine content suggests smaller interstitial spaces between the polymeric chains (i.e., higher free volume) that correspond to the increasing film density and the chitosan-DES-adenine interactions (Table 1). When adenine content increases, the formation of a denser network prevents the chitosan from swelling, reducing water vapor transmission through the film^57^. Moreover, when hydrophilic water transport is considered, adenines’s hydrophobic characteristics can not be neglected. The changes in WVTR and WVP after the addition of hydrophobic substance were previously discussed also by Bai et al.^58^, and Huang et al.^59^. Bai et al.^58^ reported antioxidative active packaging based on carboxymethyl chitosan (CMCS) with different amounts of quercetin. They noted that the addition of hydrophobic quercetin caused an increase in WVP from 15.6 10^–11^ g m m^−2 ^Pa^−1^ h^−1^ for CMCS to 18.18 10^–11^ g m m^−2^ Pa^−1^ h^−1^ for CMCS-quercetin III samples with 7.5 wt.% quercetin content. Huang et al.^59^ prepared kafirin(KF)-quercetin (KQ) films for packaging cod (Gadus morhua) fillets during cold storage at 4 °C. The addition of quercetin significantly decreased the WVP of the film from 1.84 ± 0.05 (KF film) to 1.46 ± 0.07 g mm m^-2^ h^−1^ KPa^−1^. However, the observed reduction was assumed to be very low. The Authors explained this phenomenon as an effect of possible interactions between hydroxyl and carbonyl groups in quercetin with the kafirin resulting in reduced water absorption sites.

The polysaccharide-based films’ poor water barrier properties (especially under high humidity conditions) are well-known. WVTRs of tested films are 3–100 times greater than those of conventional films made from synthetic polymers^53^, e.g., poly(L-lactic acid) (WVTR = 8.75 g m^-2^ h^-1^, 90% RH, T = 25 °C, 0.25 mm thickness) or LDPE (WVTR = 0.32 g m^−2^ h^−1^, 100% RH, T = 38 °C, 0.76 mm thickness). Thus, further modification is needed to reduce the tested films' water vapor transport properties.

It was experimentally proved that the oxygen transport represented by OTR values (Table 1) was reduced after adding adenine to the Ch-DES film. Generally, chitosan-based films constitute a good barrier against the permeation of oxygen. According to the previous findings^60^, the chitosan-based films exhibit oxygen permeability (1.6 10^–5^ cm^3^ m^−2^ day^−1^ atm^−1^) similar to the commercially available ethylene vinyl alcohol copolymer films or polyvinylidene chloride (PVDC). In contrast, films based on synthetic polymers (PET, polyethylene, etc.) exhibit higher OTR values than chitosan films^54,60,61^. As DES addition reduces the OTR values^20^, the adenine incorporation exacerbates this effect. Introducing up to 3 wt.% of adenine to the chitosan-DES film induced a significant decrease in the OTR values up to ca. 70%. It can be suggested that the OTR of the Ch-DES-A films decreased with an increasing adenine concentration due to the microstructural changes in the film. This result could be caused by the decrease in the chitosan matrix’s free volume by adding the adenine, as mentioned earlier. The results are in accordance with the other literature reports^62,63^. Cerqueira et al.^62^ prepared several chitosan-based films plasticized with glycerol or sorbitol, and hydrophobic corn oil. They found that in each case addition of oil reduces the OTR values. The Authors attributed the observed changes mainly to the differences in the internal film structure.

Similarly, Zhong et al.^63^ proved that oxygen permeability strongly depends on the interactions between the polymer matrix and the permeating gas. The improvement of oxygen barrier properties (OTR reduction) of chitosan/cassava starch/gelatin blends plasticized with glycerol increased with the increase of cassava starch and gelatin due to the formation of intermolecular hydrogen bonds between NH_4_^+^ of chitosan and gelatin backbone and OH^-^ of cassava starch. The enhanced molecular interaction resulted in a compact structure and low permeable film.

An industry rule of thumb is that a material is considered a “high oxygen barrier” if its OTR is less than 0.64 cm^3^ m^−2^ h^−1^. The HDPE and LDPE packages are characterized by^64^ ca. 95–129 and 290–350 cm^3^ m^−2^ h^−1^ OTR values (23 °C, 0% RH, ASTM D3985 with OxTran 2/21 (MOCON, Minneapolis, US)), respectively, while highly oxygen barrier ethylene vinyl alcohol by 0.0033—0.008 cm^3^ m^−2^ h^−1^ (23 °C, 0% RH, ASTM D3985 with OxTran 2/21 (MOCON, Minneapolis, US)). The very low oxygen permeability of 1.6 g m^−2^ h^−1^ of Ch-DES-A3 film suggests the promising applications of adenine-loaded chitosan film in food packaging.

### Antioxidant activity

Antioxidant properties, especially radical scavenging activities, and hydrogen peroxide radical scavenging, are significant due to free radicals' deleterious role in foods and biological systems. The oxidation of food makes it unsuitable for consumption. For this reason, novel packages are developed, representing both classic package features and preventing food spoilage. It is especially crucial when the packaging material can be consumed together with food, thus bringing added antioxidative value to the other food components.

The possible antioxidant activity of the films was measured by two methods, DPPH and H_2_O_2_. The application of two methods to evaluate the antioxidant activity has been recommended to better understand the new materials’ antioxidant properties.

#### DPPH radical scavenging assay

**Figure 7:**
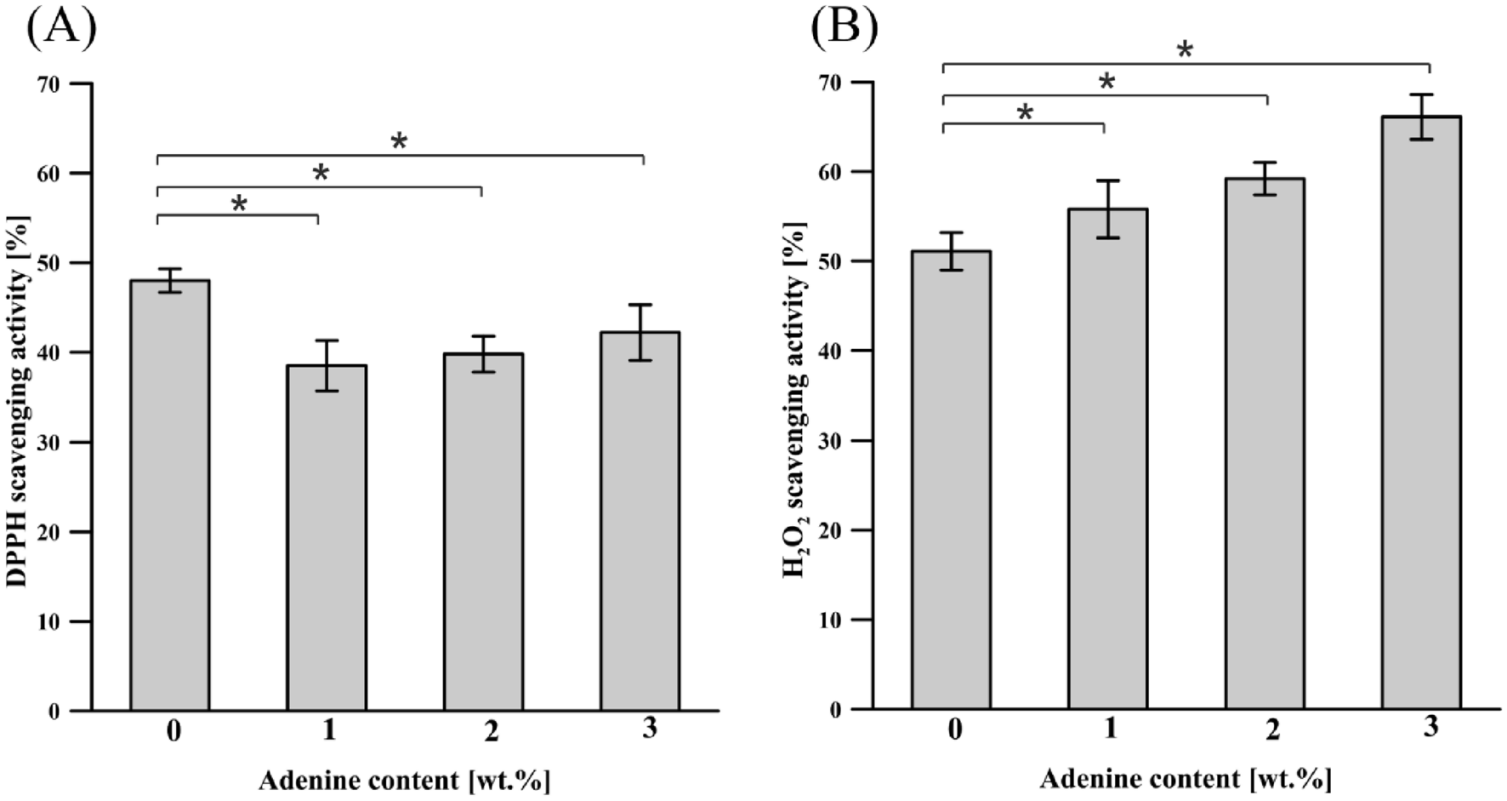
DPPH scavenging activity (**A**) and hydrogen peroxide scavenging activity (**B**) of chitosan-DES films incorporated with adenine (values with asterisks * represent significant differences (*p* < 0.05) relative to the Ch-DES).

DPPH radical scavenging assay has been widely used to test the ability of compounds to act as free radical scavengers and, thus, to evaluate the antioxidant activity of chitosan films. This assay is based on the ability of DPPH, a stable free radical, to be quenched and decolorized in the presence of antioxidants, resulting in reduced absorbance values^65^. The radical scavenging activity of chitosan-DES films with and without incorporated adenine was determined and is given in Fig. 7A. The Ch-DES control film showed a free radical scavenging activity of 48.9 ± 1.6%. It may be because that free radicals can react with the residual free amino (NH_2_) groups of chitosan to form stable macromolecule radicals, and the NH_2_ groups can form ammonium (${\mathrm{NH}}_{3}^{+}$) groups by absorbing a hydrogen ion from the solution^66,67^. It was found that citric acid can also act as an antioxidant and is very effective in retarding the oxidative deterioration of lipids in foods^67^. Incorporating adenine into Ch-DES only slightly changes the free radical scavenging activity. The free radical scavenging activity slightly decreases when 1 wt.% of adenine is reached and then negligibly increases with adenine content. Ch-DES films incorporated with 1, 2, and 3 wt.% of adenine exhibited 38.5 ± 2.8%, 39.8 ± 2.0, and 42.2 ± 3.1% DPPH scavenging activity, respectively.

#### Hydrogen peroxide radical scavenging assay

Although H_2_O_2_ is not a free radical, H_2_O_2_ is very harmful to cells because it may cross biological membranes and can be a substrate of the highly reactive hydroxyl radical by a Fenton reaction^36,68^. Avoiding H_2_O_2_ from food contact becomes an essential part of food packaging. The inhibition of hydrogen peroxide radical by Ch-DES films incorporated with adenine at varying concentrations is shown in Fig. 7B. The chitosan film with choline chloride-citric acid mixture showed a hydrogen peroxide radical scavenging activity of 51.1 ± 2.1%, which was higher than that reported by Priyadarshi et al.^42^ for citric acid crosslinked and glycerol plasticized chitosan film (29.80 ± 0.57%). The scavenging capacities of chitosan films incorporated with adenine increased with increasing adenine concentration, and the results showed that inhibition of H_2_O_2_ varied from 55.8 ± 3.2% to 66.1 ± 2.5%.

Thus, even if adenine is not known as an antioxidant, it slightly enhances the antioxidative properties of chitosan-DES films. The ANOVA analysis proved that the differences in scavenging activity between the Ch-DES and Ch-DES-A films are statistically significant (*p* < 0.05).

### Biological activity

#### Antimicrobial properties

**Figure 8:**
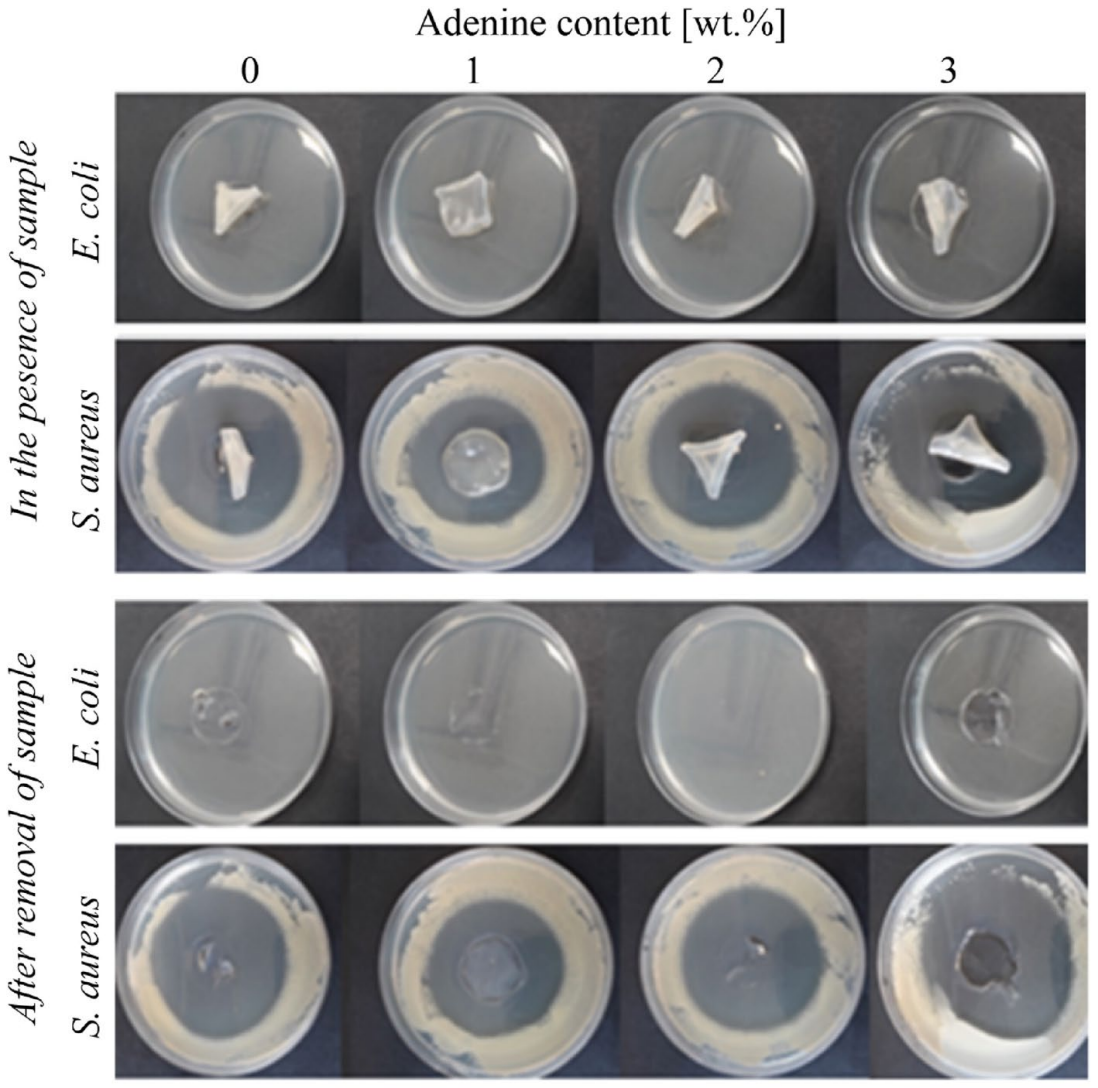
Bacterial growth and size of the inhibition zone in the presence of samples and after sample removal.

The results of the bactericidal properties of chitosan membranes with adenine are given in Table 2 and Fig. 8.

**Table 2:** Size of the growth inhibition zones [mm] of *E. coli and S. aureus*.

Adenine content [wt.%]	Size of bacterial growth inhibition zones [mm]	Bacterial growth on nutrient medium for working sample	Rating
*E. coli*			
0	> 1; ∞	Lack	Good effect
1	> 1; ∞	Lack	Good effect
2	> 1; ∞	Lack	Good effect
3	> 1; ∞	Lack	Good effect
*S. aureus*			
0	20 ± 1	Lack	Good effect
1	15 ± 1	Lack	Good effect
2	19 ± 1	Lack	Good effect
3	17 ± 1	Lack	Good effect

A good effect is required for antimicrobial treatment, obtained for gram-negative and gram-positive bacteria described in the method. There was no *E. coli* observed for all the tested films, while different sizes of inhibition zones were noted for *S. aureus* (in the range between 15 and 20 mm) (Fig. 8, Table 2). The results indicated that adding DES to chitosan films results in a film of bactericidal properties. The effectiveness of DES addition to the antimicrobial properties of chitosan materials, was already shown by others. Yu and coworkers^28^ found excellent antibacterial properties against *E. coli* and *S. aureus* of chitosan films plasticized with choline-based DESs containing acetylsalicylic acid, malonic acid, and lactic acid.

Similarly, Zhang et al.^47^ showed good antibacterial activity against *S. aureus* and *E. coli* of chitosan-lignin containing citric acid, adipic acid, and betaine. The effectiveness of DES as an antimicrobial agent was already discussed by Rachmaniah et al.^69^, Radosevic et al.^70^, and others^71^. It was indicated that the factors affecting the bacterial membrane are still unknown. Adding adenine only slightly reduces the antimicrobial activity, and within the tested adenine content, the antimicrobial effectiveness is comparable. The observed effect is probably due to the low antimicrobial characteristics of adenine itself^72^.

#### Ames test

**Figure 9:**
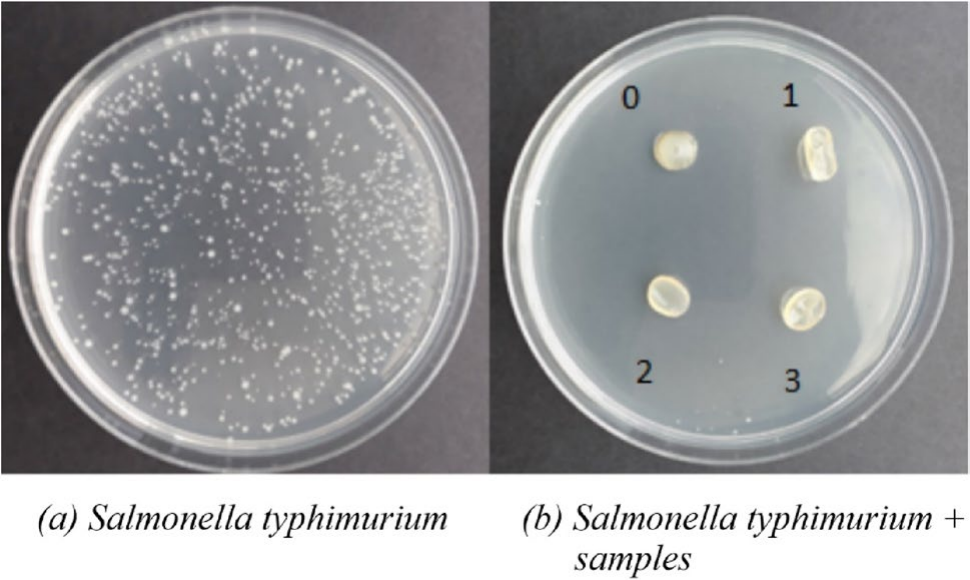
Ames test: (**a**) control—a culture of *Salmonella typhimurium* (reverse control), (**b**) tested Ch-DES-A samples in the presence of the *Salmonella typhimurium* strain (induction of mutation with tested polymer materials). Numbers refer to the adenine content in Ch-DES films.

Figure 9 shows the testing results providing information about polymeric films' mutagenicity.

The obtained results prove the lack of mutagenicity of the tested samples. In addition, this analysis indicates that the tested samples show bactericidal properties against the *Salmonella typhimurium* strain.

The results indicated that DES and adenine added to chitosan biopolymeric film do not cause biopolymer mutagenicity. It is a positive finding as DES exhibits negligible mutagenic character^73^, ascribed to the hydrogen bonds formed during its formation and the delocalized charges.

## Conclusions

The plasticized chitosan-DES films with adenine have been developed and formed into flat polymeric films. It has been proved that the materials are still dense and non-porous after adenine addition to Ch-DES. The progressive phase separation increased the water vapor permeability by 215%, which is lower when the adenine content reaches higher values (increases of 175%). Oppositely, oxygen transmission is reduced to 2.1–1.6 gm^−2^ h^−1^, and the OTR is lower than commercial HDPE and LDPE packages. Thus, Ch-DES-A films reduce the possible unfavorable food oxidation processes. The DPPH and H_2_O_2_ radical scavenging test proved the stated hypothesis that the presence of adenine and its content affects the antioxidative properties of the pristine chitosan-DES material, even if the final effect is not substantial. The changes in physicochemical and antimicrobial properties allow the classifying of the Ch-DES-A films to the active packaging. What is crucial, adenine addition unfavorably changes the mechanical properties, an essential parameter of package material. However, the measured values are still within the typical food packaging plastics range. Even if less elastic than Ch-DES (*E*_*b*_ = 146.4%), the three-component films are still more elastic (*E*_*b*_ = 35.8%) than neat chitosan. Finally, it can be stated that the developed Ch-DES-A films are an example of a material with very good biological and functional properties that are currently in demand in the packaging industry. However, these materials must still be tested in the “real-use” application conditions. The improved antioxidative properties, usage of substrates being allowed as food additives, and the presence of adenine create the advantage of the Ch-DES-A materials as edible coatings, being also a source of Vitamin B4.

### Supplementary Information

Supplementary Information.

## Data Availability

The workflows and data used are available from the corresponding author upon reasonable request.
