# Supplementary material for: The adenine-modified edible chitosan films containing choline chloride and citric acid mixture
Source: Sci Rep. 2023 Aug 3;13:12629. doi: 10.1038/s41598-023-39870-4 (PMC10400631; doi:10.1038/s41598-023-39870-4)
Supplement: Supplementary file 1 — Supplementary Information. [file 41598_2023_39870_MOESM1_ESM.pdf]

The adenine-modified edible chitosan films containing choline chloride and citric acid mixture

Magdalena Gierszewska*, Ewelina Jakubowska and Agnieszka Richert

* Correspondence: mgd@chem.umk.pl; Tel.: +48-56-611-45-24; Fax: +48-56-611-45-26

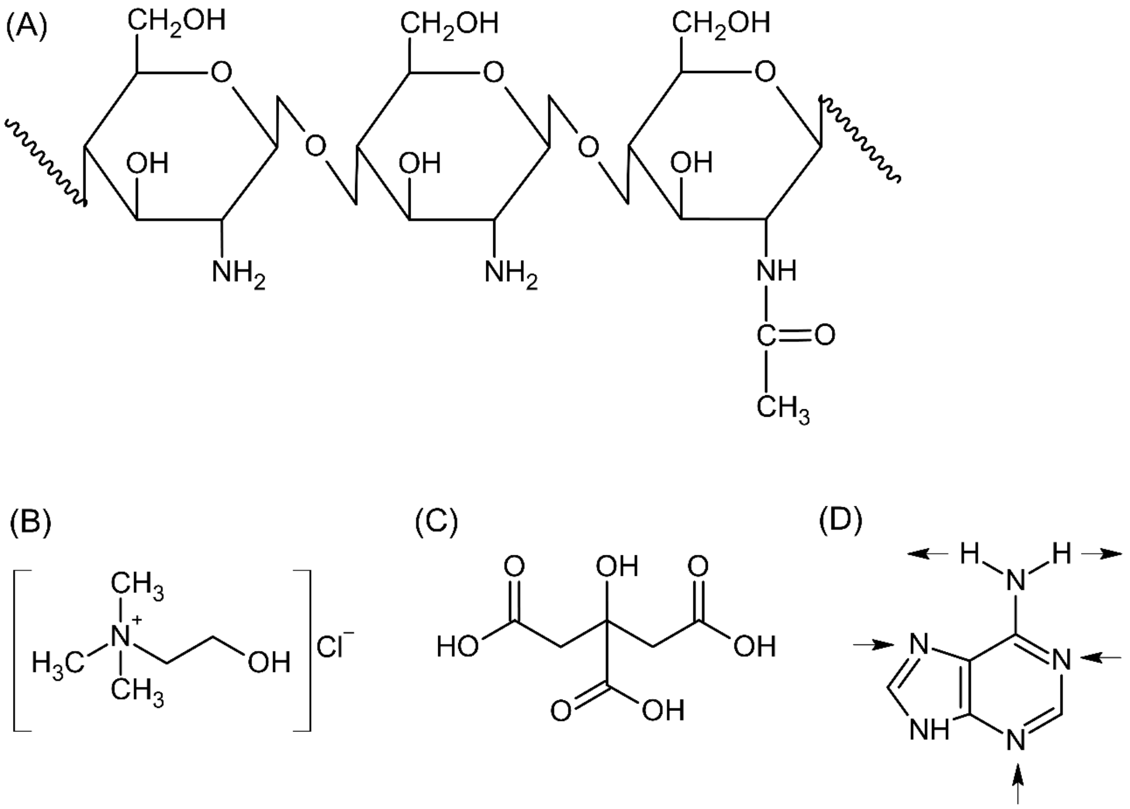

Figure S1. Chemical structure of (A) chitosan, (B) choline chloride, (C) citric acid, (D) adenine (arrow denotes atoms acting as an H-donor or H-acceptor)

The adenine-modified edible chitosan films containing choline chloride and citric acid mixture

Magdalena Gierszewska*, Ewelina Jakubowska and Agnieszka Richert

* Correspondence: mgd@chem.umk.pl; Tel.: +48-56-611-45-24; Fax: +48-56-611-45-26

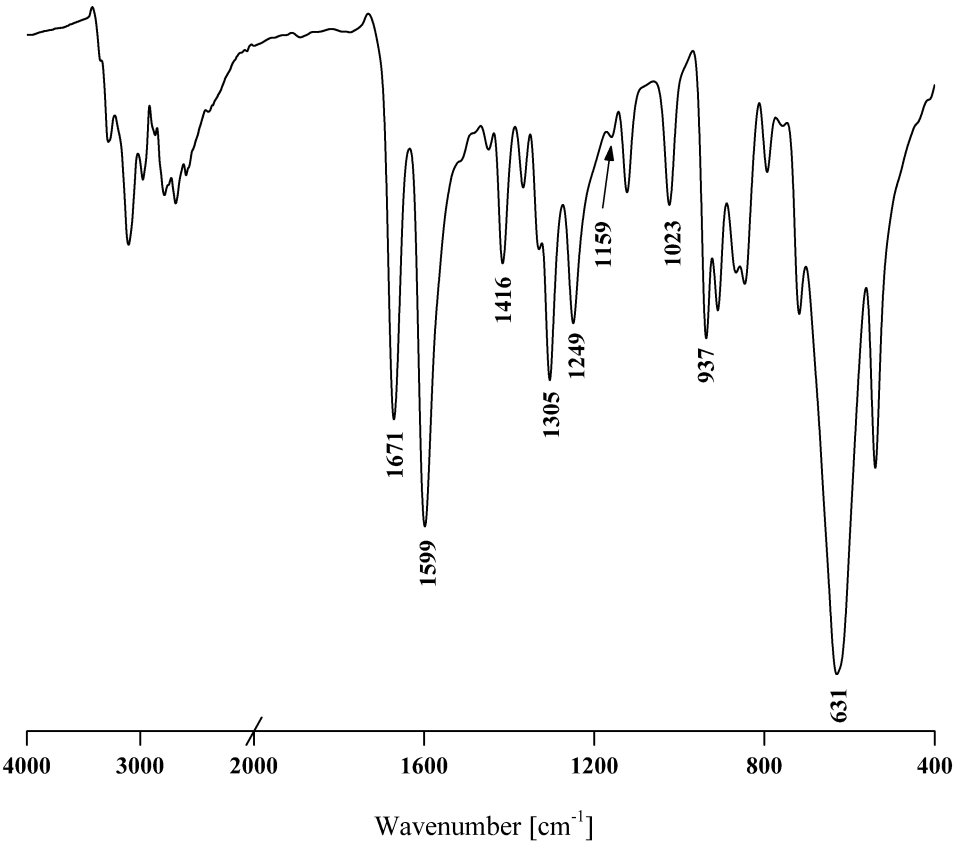

Figure S2. FTIR spectrum of adenine

The adenine-modified edible chitosan films containing choline chloride and citric acid mixture

Magdalena Gierszewska*, Ewelina Jakubowska and Agnieszka Richert

* Correspondence: mgd@chem.umk.pl; Tel.: +48-56-611-45-24; Fax: +48-56-611-45-26

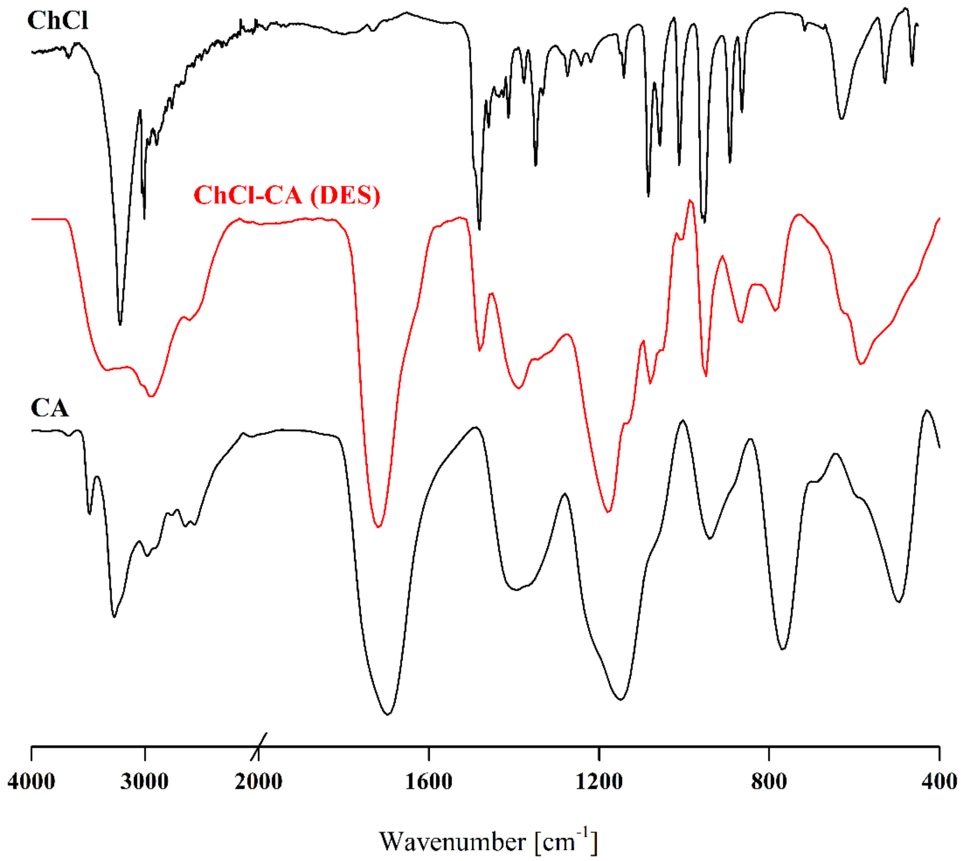

Figure S3. FTIR spectra of deep eutectic solvent (DES, ChCl-CA equimolar mixture) and its neat components (choline chloride and citric acid)

The adenine-modified edible chitosan films containing choline chloride and citric acid mixture

Magdalena Gierszewska*, Ewelina Jakubowska and Agnieszka Richert

* Correspondence: mgd@chem.umk.pl; Tel.: +48-56-611-45-24; Fax: +48-56-611-45-26

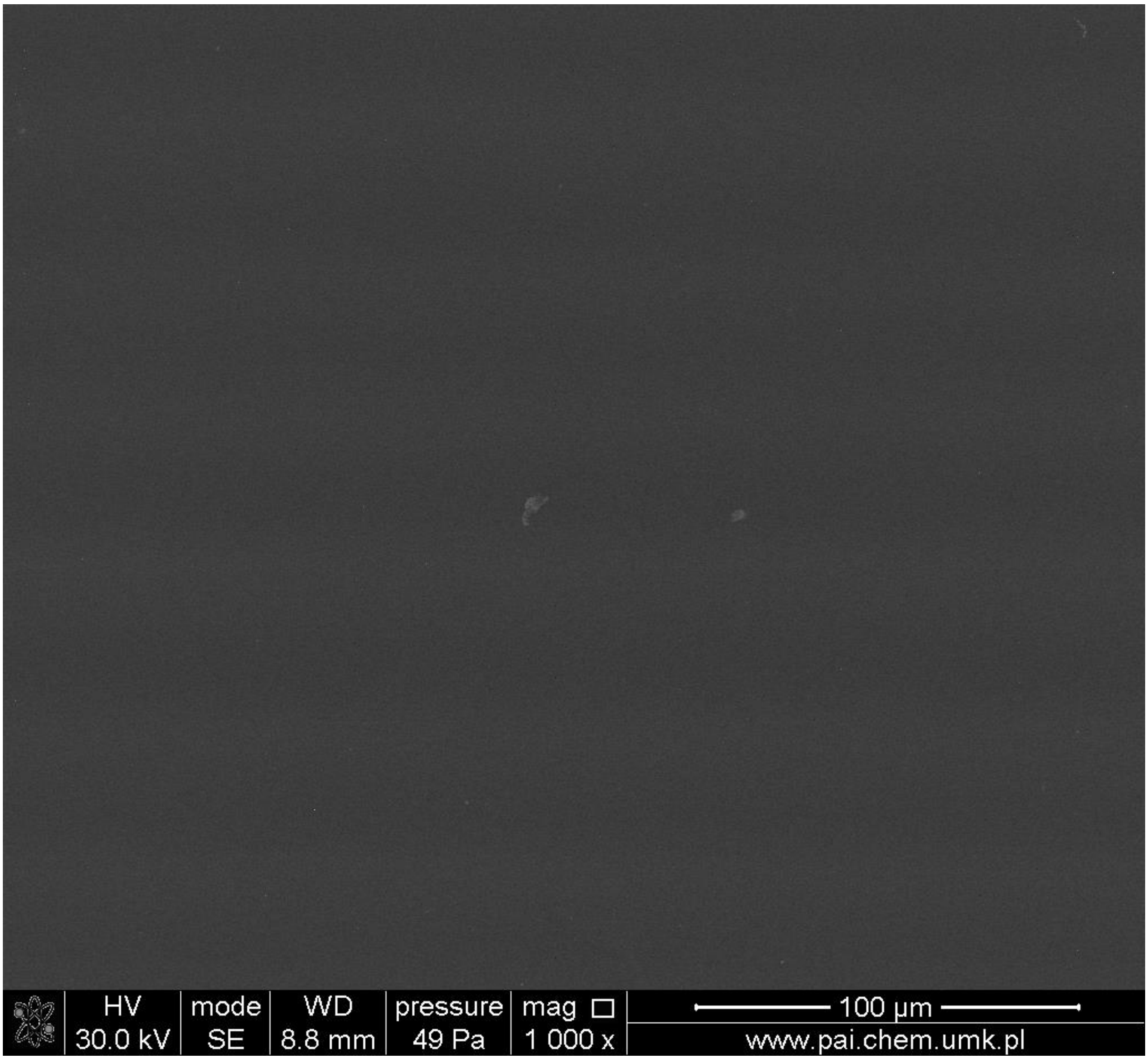

Figure S4a. SEM image of the Ch film surface (1000x)

The adenine-modified edible chitosan films containing choline chloride and citric acid mixture

Magdalena Gierszewska*, Ewelina Jakubowska and Agnieszka Richert

* Correspondence: mgd@chem.umk.pl; Tel.: +48-56-611-45-24; Fax: +48-56-611-45-26

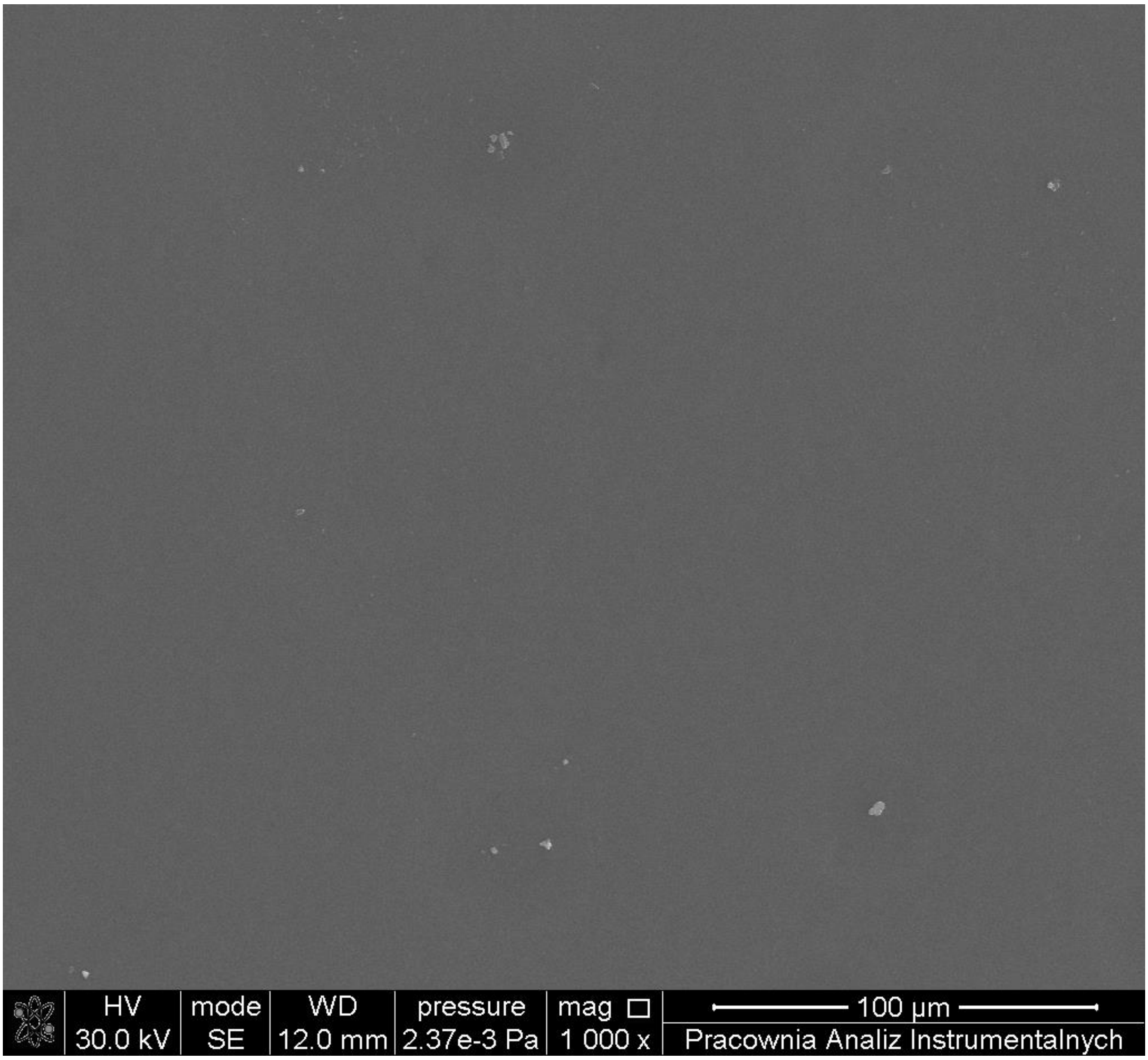

Figure S4b. SEM image of the Ch-DES film surface (1000x)

The adenine-modified edible chitosan films containing choline chloride and citric acid mixture

Magdalena Gierszewska*, Ewelina Jakubowska and Agnieszka Richert

* Correspondence: mgd@chem.umk.pl; Tel.: +48-56-611-45-24; Fax: +48-56-611-45-26

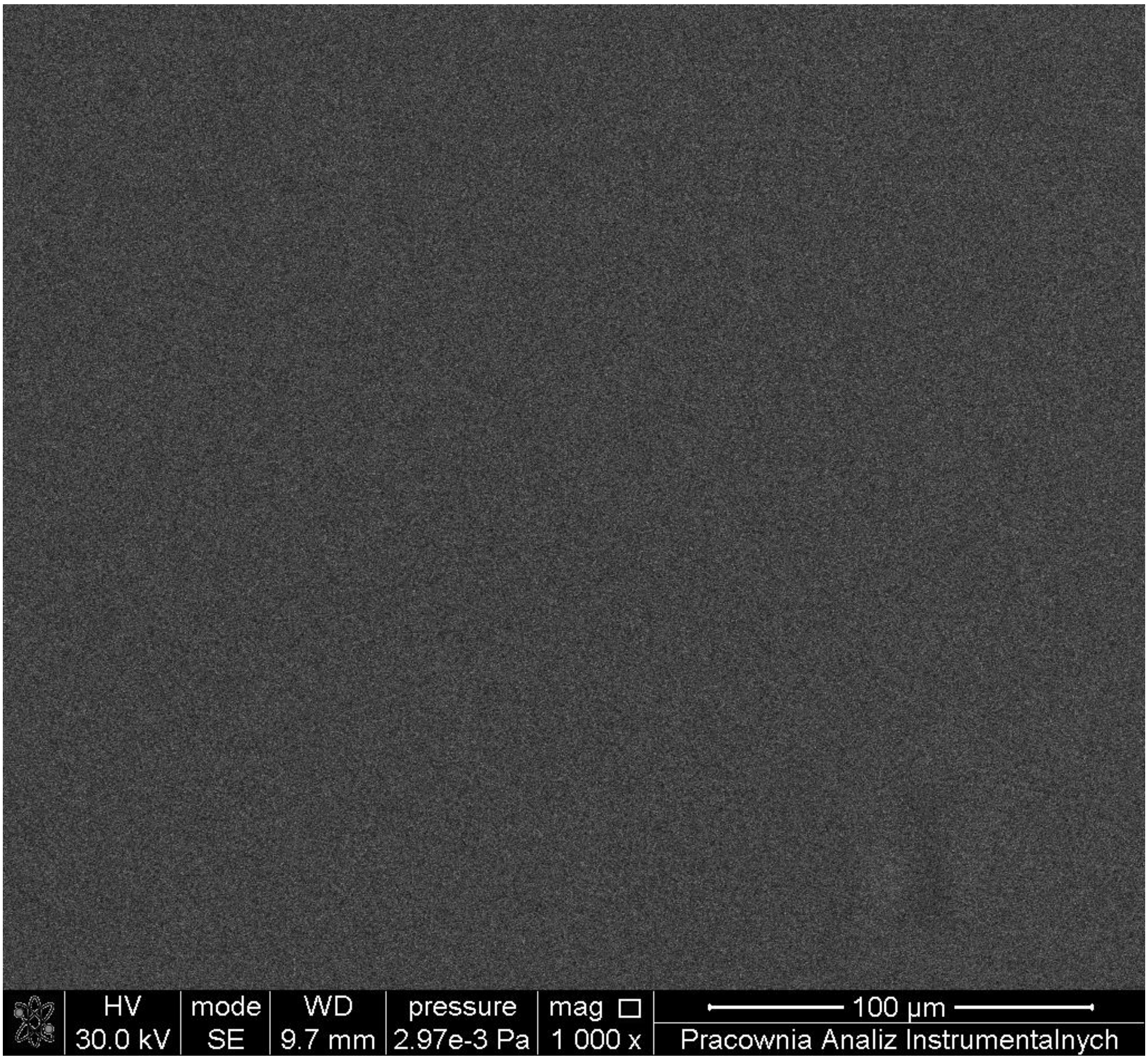

Figure S4c. SEM image of the Ch-DES-A1 film surface (1000x)

The adenine-modified edible chitosan films containing choline chloride and citric acid mixture

Magdalena Gierszewska*, Ewelina Jakubowska and Agnieszka Richert

* Correspondence: mgd@chem.umk.pl; Tel.: +48-56-611-45-24; Fax: +48-56-611-45-26

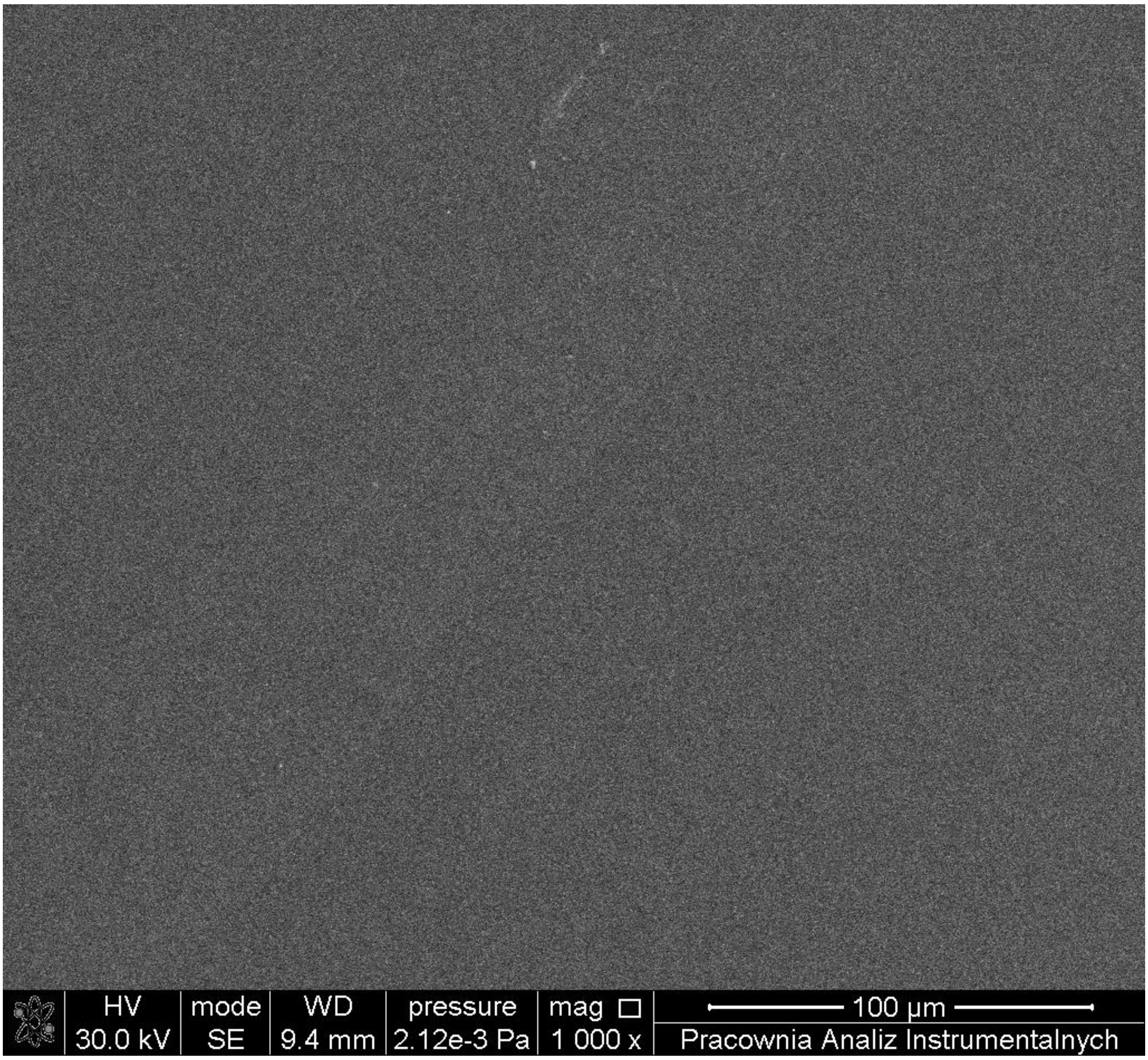

Figure S4d. SEM image of the Ch-DES-A1 film surface (1000x)

The adenine-modified edible chitosan films containing choline chloride and citric acid mixture

Magdalena Gierszewska*, Ewelina Jakubowska and Agnieszka Richert

* Correspondence: mgd@chem.umk.pl; Tel.: +48-56-611-45-24; Fax: +48-56-611-45-26

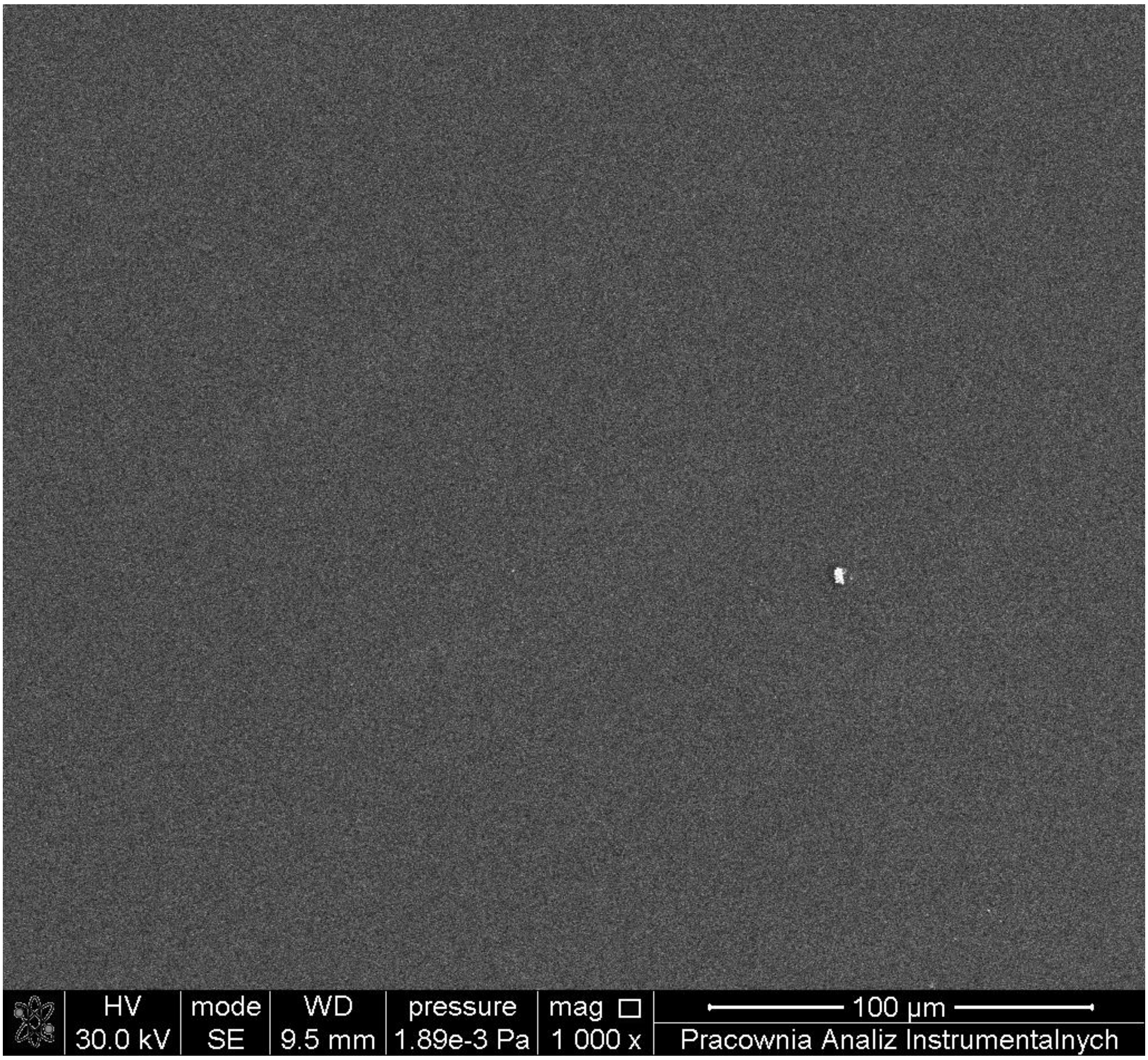

Figure S4e. SEM image of the Ch-DES-A1 film surface (1000x)

The adenine-modified edible chitosan films containing choline chloride and citric acid mixture

Magdalena Gierszewska*, Ewelina Jakubowska and Agnieszka Richert

* Correspondence: mgd@chem.umk.pl; Tel.: +48-56-611-45-24; Fax: +48-56-611-45-26

Table S1. Antibacterial effect of antibacterial treatment (ISO 20645, 2006)

Braking zone [mm] The average value of rise	Growth [a]	Description	Rating
>1	lack	Inhibition zone above 1, no increase [b]	
1 - 0	lack	Growth inhibition zone up to 1, no growth [b]	Good effect
0	lack	No braking zone, no increase [c]	
0	weak	Lack of braking zones, only some colonies limited growth almost completely stopped [d]	Limited Efficiency
0	average	No braking zone, height reduced to half compared to control [e]	
0	powerful	Lack of braking zones, the absence of a reduction in growth compared to the control, or only a slight reduction in growth	Insufficient effect

[a] Bacterial growth on the medium under the working sample.

[b] The dynamometer range should only be partially taken into account in the calculations. An increase in the braking zone may be due to excess active substance or unevenness of the substance in the article.

[c] Lack of growth with a simultaneous lack of braking zone can be considered a good effect. A braking zone may not be possible due to limited diffusion.

[d] "Almost as good as lack of growth" - an indication of limited efficiency.

[e] Limited bacterial growth density means both the number of colonies and the diameter of the colonies.

The adenine-modified edible chitosan films containing choline chloride and citric acid mixture

Magdalena Gierszewska*, Ewelina Jakubowska and Agnieszka Richert

* Correspondence: mgd@chem.umk.pl; Tel.: +48-56-611-45-24; Fax: +48-56-611-45-26

Table S2. ANOVA significance analysis of the influence of the adeine content on the different chitosan-DES films' parameters (statistically significant differences versus the adenine content reflects $p < 0.05$)

Parameter	Sum of squares		df	Mean Square	F-Value	p-Value
L	Between groups	47.120	3	15.707	13.778	0.002
	Within groups	9.120	8	1.140		
	Total	56.240	11			
a	Between groups	15.363	3	5.121	219.464	<0.001
	Within groups	0.187	8	0.023		
	Total	15.549	11			
b	Between groups	29.062	3	9.687	91.535	<0.001
	Within groups	0.847	8	0.106		
	Total	29.909	11			
ΔE	Between groups	54.007	3	18.002	24.083	<0.001
	Within groups	5.980	8	0.747		
	Total	59.987	11			
Chroma	Between groups	52.989	3	17.663	83.120	<0.001
	Within groups	1.700	8	0.213		
	Total	54.689	11			
Hue	Between groups	15.603	3	5.201	0.297	0.827
	Within groups	140.200	8	17.525		
	Total	155.802	11			
Opacity	Between groups	0.849	3	0.283	230.940	<0.001
	Within groups	0.020	16	0.001		
	Total	0.869	19			
Thickness	Between groups	0.000	3	0.000	30.145	<0.001
	Within groups	0.000	8	0.000		
	Total	0.000	11			
Density	Between groups	0.307	3	0.102	174.734	<0.001
	Within groups	0.005	8	0.001		
	Total	0.311	11			
WVTR	Between groups	835.709	3	278.570	91.660	<0.001
	Within groups	24.313	8	3.039		
	Total	860.023	11			
WVP	Between groups	14.569	3	4.856	104.065	<0.001
	Within groups	0.373	8	0.047		
	Total	14.943	11			
OPR	Between groups	5.963	3	1.988	10.415	0.004
	Within groups	1.527	8	0.191		
	Total	7.489	11			
DPPH	Between groups	264.629	3	88.210	15.794	<0.001

	Within groups	89.360	16	5.585		
	Total	353.989	19			
H_2O_2	Between groups	357.313	3	119.104	19.633	<0.001
	Within groups	48.533	8	6.067		
	Total	405.847	11			
